# Increased trial-to-trial similarity and reduced temporal overlap of muscle synergy activation coefficients manifest during learning and with increasing movement proficiency

**DOI:** 10.1038/s41598-024-68515-3

**Published:** 2024-07-31

**Authors:** Paul Kaufmann, Willi Koller, Elias Wallnöfer, Basilio Goncalves, Arnold Baca, Hans Kainz

**Affiliations:** 1 Department of Biomechanics, Kinesiology and Computer Science in Sport, Centre for Sport Science and University Sports, University of Vienna, Auf Der Schmelz 6a (USZ ||), 1150 Vienna, Austria; 2 Neuromechanics Research Group, Centre for Sport Science and University Sports, University of Vienna, Auf Der Schmelz 6a, 1150 Vienna, Austria

**Keywords:** Computational neuroscience, Motor control, Learning and memory, Neurophysiology

## Abstract

Muscle synergy analyses are used to enhance our understanding of motor control. Spatially fixed synergy weights coordinate multiple co-active muscles through activation commands, known as activation coefficients. To gain a more comprehensive understanding of motor learning, it is essential to understand how activation coefficients vary during a learning task and at different levels of movement proficiency. Participants walked on a line, a beam, and learned to walk on a tightrope—tasks that represent different levels of proficiency. Muscle synergies were extracted from electromyography signals across all conditions and the number of synergies was determined by the knee-point of the total variance accounted for (tVAF) curve. The results indicated that the tVAF of one synergy decreased with task proficiency, with the tightrope task resulting in the highest tVAF compared to the line and beam tasks. Furthermore, with increasing proficiency and after a learning process, trial-to-trial similarity increased and temporal overlap of synergy activation coefficients decreased. Consequently, we propose that precise adjustment and refinement of synergy activation coefficients play a pivotal role in motor learning.

## Introduction

The underlying mechanisms by which the central nervous system governs movement control and adapts during skill acquisition are still not fully understood. Muscle synergy analyses are widely utilized in the field of motor control to model how the central nervous system coordinates movements^1–3^. In simple terms, muscle synergies refer to groups of co-active muscles, known as synergy weights, that are recruited by activation coefficients. While synergy weights are assumed to be spatially structured in the spinal cord, activation coefficients represent time-dependent control inputs from supraspinal areas^1,3^. In accordance with Bernstein’s levels of movement construction^4,5^, muscle synergies simplify the complex coordination of the numerous muscles in the human body by controlling the activation of a limited number of spatially fixed, and temporally independent synergy weights, rather than controlling each muscle individually.

Over the last two decades, researchers have studied muscle synergies extracted from electromyography recordings in both healthy and pathological populations performing various tasks. These studies have demonstrated that similar synergy weights are recruited in different movements. So-called shared synergies are likely to occur when different movement tasks involve common mechanical subtasks^6^. For instance, shared synergies have been found between walking and cycling^7^, walking and slipping^6^, walking and standing reactive balance tasks^8^, stepping and non-stepping postural behaviors^9^, seated and standing cycling^10,11^, and overground and treadmill running^12^. The identification of common synergies across different movements provides additional support for the concept of spatially fixed synergy weights. Furthermore, studies on the neuronal organization within the spinal cord in animals have provided evidence in support of the muscle synergy hypothesis^13–15^. To describe the complexity of motor control, the total variance accounted for (tVAF) by a given number of synergies and the number of needed synergies are widely used parameters. For example, fewer synergies and higher tVAF—indicating lower motor complexity—have been found in people with cerebral palsy^16,17^ and stroke^18,19^ compared to unimpaired populations, and in younger compared to older adults during walking^20,21^.

It is generally accepted that generating identical movements in successive attempts is impossible due to the inherently noisy nervous system^22^. This noise can originate from either the central nervous system, through movement planning, or from peripheral structures, such as force production by muscles. In 2017, Dhawale et al.^23^ conducted a review on movement variability in motor learning. They concluded that variability in the planning space is more likely a feature of the plasticity of the motor system that drives motor learning, rather than unwanted noise. Furthermore, trial-to-trial similarity of movements increases with increasing task proficiency^23–26^, aligning with the principles of reinforcement learning^23^. Based on reinforcement learning theory a system learns new behavior through a process of trial and error^27^. Motor commands that lead to favorable outcomes (i.e., successful execution of a movement task) are repeated, reinforced, and refined in subsequent attempts. Wu et al.^24^ conducted a study in which participants were trained to replicate a curve shape using hand trajectories in a reaching task. They found that individuals with higher kinematic variability prior to training showed faster rates of learning. Therefore, it appears that variability during the learning process increases the likelihood of finding the optimal motor command.

To date, only a few studies have examined the role of muscle synergies in movement learning. For instance, Sylos-Labini et al.^28^ compared synergy activation coefficients in walking across different age groups, from neonates to adults. They observed an increase in trial-to-trial similarity of activation coefficients during locomotor development. Consistent with a previous study on locomotor development^29^, the authors additionally showed that motor complexity and the number of synergies increased with age. Among young adults, an increase in trial-to-trial similarity of activation coefficients was highly correlated with improvements in bowling scores over three sessions^30^. Sawers et al.^31^ found that professional ballet dancers had higher trial-to-trial similarity and higher beam walking proficiency compared to individuals with no dance or gymnastics experience. Additionally, dancers exhibited higher consistency within synergy weights and higher spatial distinctness between synergy weights, with high spatial distinctness indicating that each synergy weight is composed of unique muscle contributions. Similarly, dance-based rehabilitation improved the consistency and spatial distinctness of synergy weights in individuals with Parkinson’s disease^32^. All of the studies mentioned were limited either by inter-participant comparisons^28,33–36^, which neglected individual differences in motor control, or inter-session comparisons^35–37^, which neglected variations in skin–electrode impedance and electrode position. A number of studies conducted by Berger and colleagues^38–40^ sought to overcome these limitations by employing a within-participant, within-session motor learning protocol. Briefly, participants were required to utilize isometric muscle contractions in order to move a mass within a virtual environment. Subsequently, muscle combinations to control the mass were altered by modifying the mapping between recorded muscle activity and the simulated force applied to the mass. If the mapping was altered in a way that participants could accurately move the mass only by modifying the temporal activation coefficients of existing synergy weights, the rate of adaptation was faster and the success rate was higher compared to a change of mapping that would require the acquisition of new synergy weights. Consequently, the authors proposed that adaptations of activation coefficients play a pivotal role in early learning, while actual changes of synergy weights or the formation of new synergies could potentially emerge after a longer time span. Although these findings provide comprehensive information regarding skill acquisition, the experiments were limited to isometric tasks and the learning of muscle combinations, rather than learning actual movements.

To the best of our knowledge, no study has yet explored changes in muscle synergies during movement learning using a within-participant, within-session protocol. Therefore, we collected motion capture data and electromyography signals from participants performing a learning task, i.e., learn to walk on a tightrope, which could be learned within one data collection session. Participants completed two additional walking tasks: walking on a line, which is an easy task with high movement proficiency, and walking on a beam, which was a less common but still manageable task for participants. We investigated whether there were differences in motor complexity (quantified with the tVAF of synergies), trial-to-trial similarity of activation coefficients, and temporal overlap of activation coefficient between (1) an early and a late stage of a learning process (i.e.: learning to walk on a tightrope), as well as between (2) common and less common movement tasks (i.e.: walking on a line vs a beam vs a tightrope)—addressing movement proficiency. Additionally, we investigated whether the contribution of synergy weights to a movement task changes during learning or with different proficiency levels. The study primarily focused on muscle synergies, but also analyzed trial-to-trial similarity of joint angles to gain a more comprehensive understanding of motor learning. We hypothesized that motor complexity, trial-to-trial similarity of synergy activation coefficients and joint angles would (1) increase during learning and (2) be higher for more common movements. In contrast, a decreased temporal overlap of activation coefficients was expected (1) after learning and (2) with higher task proficiency. Our study's findings will provide new insights into motor control adaptations during learning processes.

## Materials and methods

### Participants

Ten healthy participants (age: 25.2 ± 3.34 years; bodyweight: 69.9 ± 7.34 kg; height: 1.76 ± 0.09 m; body-mass-index: 22.63 ± 1.51 kg/m^2^; 6 men and 4 women) without neurological or orthopedic impairments were recruited for this study. All participants were recreational athletes from various disciplines who exercised at least twice a week and were not able to walk on a slackline or tightrope beforehand. The study was conducted in accordance with the relevant guidelines and regulations after receiving approval from the ethics committee of the University of Vienna (reference number: 00820). Written informed consent was obtained from all participants prior to data collection.

### Experimental setup and data collection

**Figure 1:**
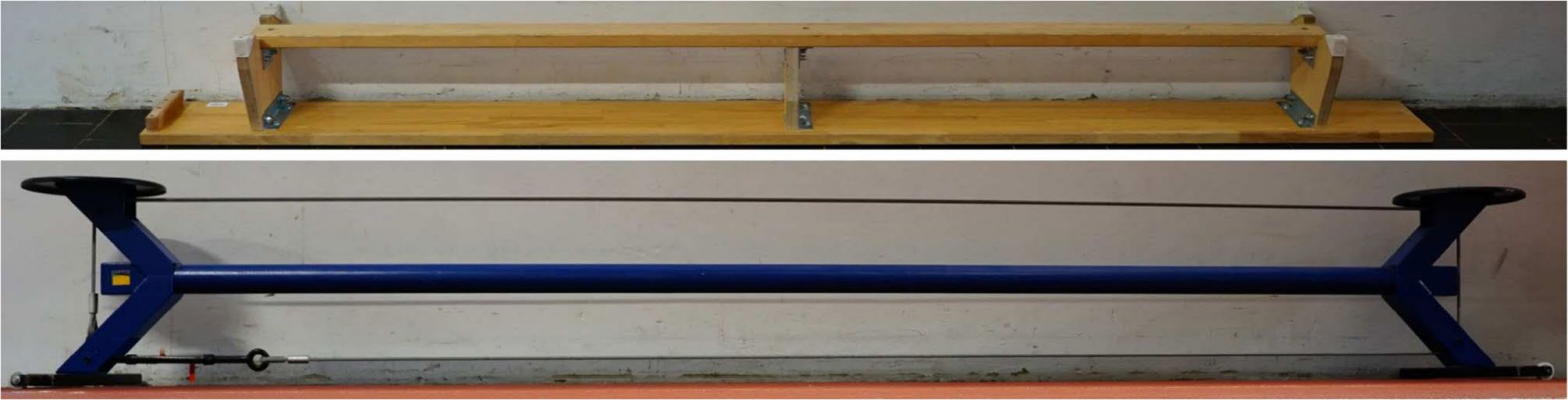
Top image shows the upside-down gymnastics bench which was used for the beam walking conditions. Bottom image shows the tightrope mounted on a rack between two platforms.

Each participant performed three different walking tasks: (a) walking along a line taped on the ground (LINE; length: 310 cm; width: 1.4 cm); (b) walking on a beam (BEAM; length: 341.5 cm; width: 10 cm; height: 28.5 cm) and (c) walking over a tightrope (TIGHTROPE; length: 363 cm; diameter: 0.9 cm; height: 52 cm) spanned between two platforms (Fig. 1). The process of learning to walk on the tightrope was divided into two stages: TRfail and TRsucc. TRfail consisted of the first five attempts, during which participants were able to complete at least one full gait-cycle of the right leg but were unable to maintain balance over the entire tightrope. TRsucc included the attempts in which participants successfully walked over the tightrope in four out of five consecutive attempts. A successful attempt was defined as walking the entire length of the tightrope and maintaining balance on the second platform. If a participant successfully balanced over the entire tightrope in two out of the first five attempts, the task’s difficulty was increased with visual constraints. This was done by either placing an eye-patch over their left eye or, if they succeeded in two out of the first five attempts with an eye-patch, by closing both eyes. The conditions were recorded in the following order: (1) LINE-walking (startLINE), (2) BEAM-walking (startBEAM), (3) the start of the learning process on the TIGHTROPE (TRfail) until (4) the end of the learning process (TRsucc), (5) BEAM-walking (endBEAM), and (6) LINE-walking (endLINE). To ensure consistent visual constraints across tasks for later comparisons, five trials with opened eyes, an eye-patch over the left eye and both eyes closed were recorded each time (start and end) for LINE and BEAM. For participants who completed the learning task (i.e., walking over the tightrope) with visual constraints, the LINE and BEAM tasks with matching visual constraints were selected for further analyses to ensure that neural pathways were equally affected in each condition. As data from the first right stance phase was further analyzed, we aimed to minimize transient accelerations at the onset step^17,20,41^ by instructing participants to start each trial with their left leg. Only the stance phase was analyzed to neglect highly variable movement times between stance and swing phases across conditions^36,42^. No additional constraints were given for pause time, step cadence, step length, or hints for walking over the tightrope, in order to facilitate self-directed learning.

Prior to the data collection, thirteen surface electromyography (EMG) sensors (eleven PicoEMG and two Mini Wave Infinity, Wave Plus wireless EMG system, Cometa, Milan, Italy) were placed on the trunk and right limb according to the Seniam guidelines (Seniam.org), and recommendations from previous studies^43–45^: tibialis anterior (tib_abt), peroneus longus (per_long), soleus, gastrocnemius medialis (gast_med), vastus lateralis (vast_lat), rectus femoris (rect_fem), biceps femoris (bic_fem), semitendinosus (sem_tend), gluteus maximus (glut_max), rectus abdominis (rect_abd), extensor obliques (ext_obli), lumbar multifidus (multifid) and erector spinae iliocostalis (erec_spin). A baseline EMG signal of several seconds was recorded while participants lay in a supine and relaxed position on a massage table. The standard Vicon Plug-in-Gait marker set (Vicon, Oxford, UK), including 21 reflective markers, was placed on the legs, pelvis and trunk of each participant^46^. The heel and toe markers were placed on the participants’ shoes, similar to Paterson et al.^47^. A 12-camera 3D motion capture system (Vicon, Oxford, UK) was used to simultaneously record marker trajectories at a sampling rate of 200 Hz, EMG data at 1000 Hz and ground reaction forces from one force plate at 1000 Hz (Kistler Instrumente, Winterthur, Switzerland). In addition, participants wore in-shoe force sensor insoles (loadsol^®^, Novel, Munich, Germany), which were used to determine stance phases. Insoles data was captured at 200 Hz (loadsol-s android application version 1.7.63) on a mobile phone (Huawei P30 Lite, Huawei, Shenzhen, China) and brought to zero level every 5–10 trials to minimize errors caused by sensor drifts. Foot contact instances were determined by custom scripts based on vertical contact forces over 30 N. Time synchronization between the insole and Vicon data was achieved by participants stepping on the force plate at the beginning of each trial. The experimental data was captured and processed using Vicon Nexus 2.12 software (Vicon, Oxford, UK). Subsequent analyses were conducted using Gnu Octave version 6.2.0^48^ and MATLAB (R2022a, Mathworks Inc., Natick, USA).

### EMG processing

Raw EMG signals were high-pass filtered at 25 Hz (4th-order Butterworth zero lag-filter) to remove movement artefacts^17,49–51^, demeaned, full-wave rectified and low-pass filtered at 7 Hz (4th-order Butterworth zero-lag filter), similar to previous gait studies^52–56^. The choice of a 7 Hz low-pass cutoff frequency was a compromise due to different stance phase duration times between the three walking tasks (Supplementary Fig. S1). After filtering, baseline noise was removed by subtracting the root-mean-square of the filtered baseline EMG signal to improve the signal-to-noise ratio^57–60^, and any resulting negative values were set to zero. Based on a visual inspection of raw and filtered EMG envelopes, trials with artefacts (e.g., movement artefacts) were removed, resulting in 4–5 remaining trials per condition. Afterwards, signals were time-normalized to 101 data points (100% of stance phase) and amplitude normalized to values between 0 and 1, where an amplitude of 1 was equal to the maximum activation amplitude of a muscle among all trials^19,21,41,42,54,61,62^.

### Synergy extraction and determining the number of synergies

For each participant, processed EMG signals of trials from all conditions were concatenated^10,63^ and muscle synergies were extracted according to the spatial/synchronous synergy model^58^. This model describes motor control of muscle activations (EMG signals) as a linear combination of fixed spatial synergy weights and time-varying activation coefficients^4,42,58^. Non-negative-matrix-factorization has been shown to be the most appropriate method for extracting muscle synergies in walking^56^. Therefore, we used the “nmf_bpas” octave function^64^, an advanced algorithm of classical non-negative-matrix-factorization^65–67^, to extract 1–12 (number of muscles − 1) muscle synergies. Instead of random inputs, the algorithm was initialized with outputs of the nonnegative single-value-decompensation with low-rank correction algorithm^68^ to improve the factorization^58,68–70^. Extracted synergy weights were normalized to 1 based on their maximum values, and activation coefficients were multiplied by the same normalization values to keep their product constant^71,72^. Detailed information on the synergy extraction procedure is provided in the Supplementary material.

The overall total variance accounted for (tVAF) among all conditions was calculated for each number of extracted synergies (1–12). It quantifies the reconstruction accuracy after the factorization, and is defined as the uncentered Pearson’s correlation in percentage^55^. To determine the number of synergies that represents motor control across all conditions (NoS), knee point analysis was employed^42,50,55,58,73^ on the overall tVAF curve. The knee-point (v) was defined as the point on the tVAF curve that exhibits the smallest angle among three adjacent points (v − 1, v, v + 1). This approach assumes that beyond the knee-point, only unstructured data or noise is explained by additional synergies^73^. The knee-point method was preferred over threshold-based methods due to its superior performance^55^ and independence from varying low-pass filter cutoff frequencies^50^. We further constrained our analysis by only determining the knee-point for synergies with a tVAF exceeding 95%. This threshold has been widely used^52,55,60,63,74–76^ and was added based the visual observation of sharp jumps in some tVAF curves, likely caused by the splitting of a synergy due to salient features^77^.

### Synergy analyses

#### Overall synergy analyses

**Figure 2:**
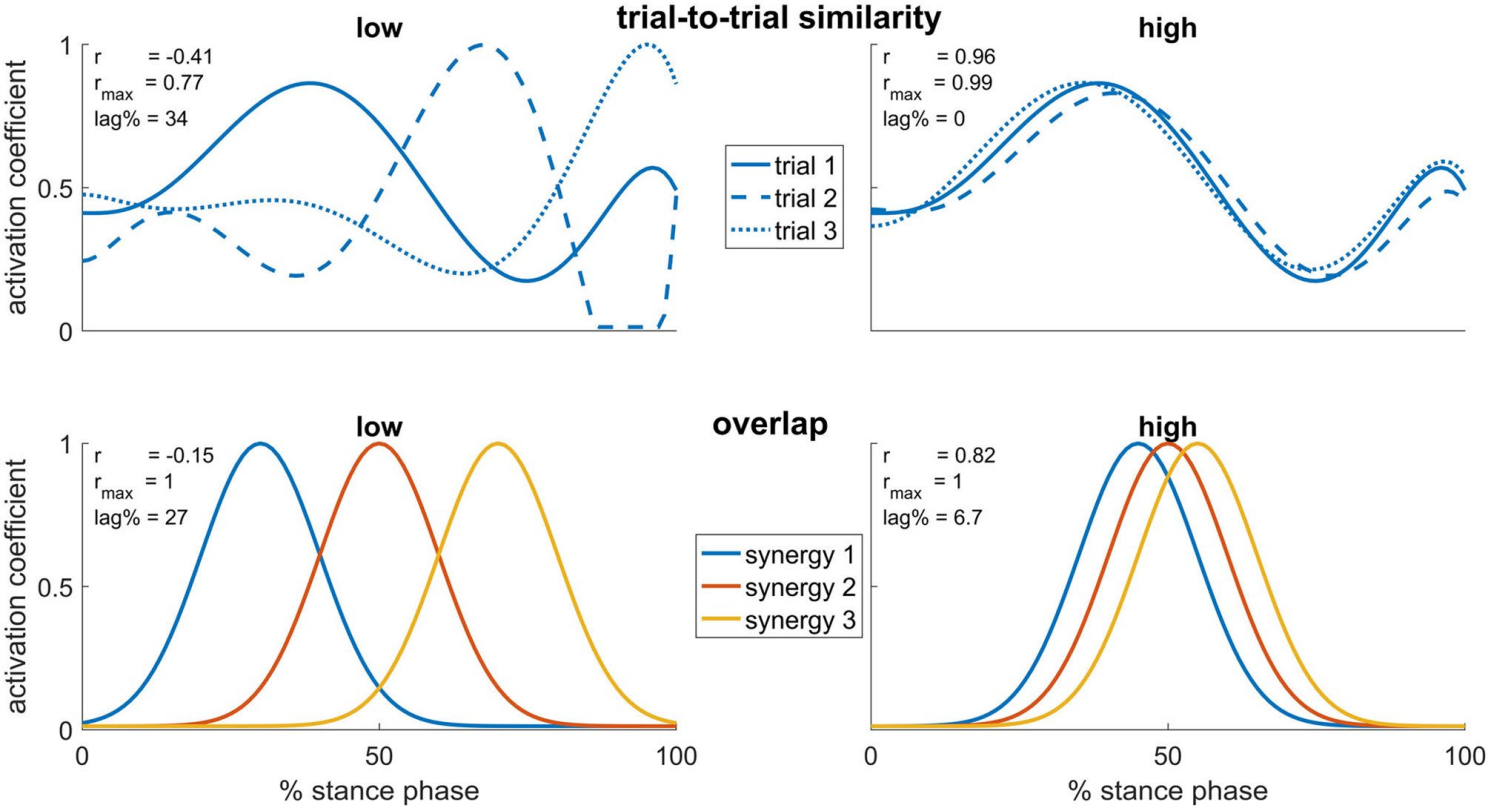
Schematic illustration depicting the two primary analyses performed on synergy activation coefficients. In the upper panel, trial-to-trial similarity was evaluated across different trials of the same synergy. In the lower panel, overlap was assessed among different synergies within the same trial. For each example, the Pearson correlation coefficient (r), the maximum value of the normalized cross-correlation coefficient (r_max_), and the lag time at which r_max_ occurred, indicating the time shift between two curves (lag% as a percentage of the stance phase), are presented. These values were calculated as the mean of each pairwise comparison among the three waveforms.

We calculated tVAF using the EMG signals, synergy weights and activation coefficients of each condition. Then, the tVAF of one synergy (tVAF1) and the tVAF at NoS (tVAFNoS) were compared across conditions to assess movement complexity (tVAF1) and the goodness of reconstruction (tVAFNoS). Overlap and trial-to-trial similarity of activation coefficients were quantified using the Pearson correlation coefficient (r), the maximum value of the normalized cross-correlation coefficient (r_max_) and the lag time at which r_max_ occurred, indicating the time shift between two curves (lag% in % of the stance phase). These parameters are widely employed in biomechanical studies to analyze waveforms^7,10,11,71,78–80^. In our study, we used all three parameters as they evaluate slightly different aspects (Fig. 2). The overlap of activation coefficients was determined by calculating the average value of all pairwise combinations of activation coefficients from different synergies within each trial for each condition. High values of r and r_max_, along with small time-shifts (lag%), indicate temporal similarity and therefore a substantial amount of overlap in synergy activation coefficients^19,21,69^. The overall trial-to-trial similarity of activation coefficients was determined by calculating the average value of all pairwise combinations of activation coefficients from different trials within each synergy for each condition. High values of r and r_max_, along with small time-shifts (lag%), indicate high trial-to-trial similarity.

#### Individual synergy analyses

In addition to evaluating the overall trial-to-trial similarity of each walking condition, we aimed to determine whether any observed differences in trial-to-trial similarity were specific to particular synergies. This necessitated the identification of similar synergy weights among participants. To classify similar synergy weights, we used k-means clustering (kmeans function in Octave—see Supplementary material), similar to recent synergy studies^28,30,34,54,81^. We computed the k-means clustering solution for a range of two to twelve clusters and repeated the process 100 times. For each repetition and each number of clusters, we calculated the average silhouette value^82^. The optimal number of clusters was then determined at the point where the maximum silhouette values plateaued, indicating small within- and high between-cluster distances^30^. Trial-to-trial similarity parameters (r, r_max_, lag%) were calculated for synergies within the same cluster for each condition. For instance, if a cluster consisted of eight synergy weights, the trial-to-trial similarity of that cluster was determined by the trial-to-trial similarity values of the eight synergies. To examine the task-specific relevance of individual synergy weights, the tVAF of each synergy was calculated for every trial. These tVAF values were then averaged across trials of the same condition for each synergy within the same cluster.

### Kinematic analyses

Joint angles were computed with OpenSim^83^ using the recently introduced addBiomechanics.org application^84^. This application uses bilevel optimization and enables to personalize musculoskeletal models and calculate joint angles in an easy and efficient way. We used the default option with the Rajagopal2015 model for human gait^85^. The joint angles were computed and then smoothened using a 6 Hz low-pass filter (4th-order Butterworth zero-lag filter) and time normalized to 101 datapoints of the stance phases. The study examined the following joint angles of the right leg and trunk: ankle plantar-/dorsiflexion, knee flexion/extension, hip flexion/extension, hip ab-/adduction, hip internal/external rotation, lumbar flexion/extension, lumbar medial/lateral bending, and lumbar internal/external rotation. The trial-to-trial similarity of each joint angle was determined by calculating the r, r_max_ and lag% values of all pairwise combinations of trials for each condition. The overall trial-to-trial similarity of joint angles was then defined as the average of these values. Additionally, correlation values were also averaged for each joint separately to assess whether similarity differs in each joint (results provided in Supplementary material).

### Statistics

We employed a two-way repeated measures ANOVA with TASK (line, beam, tightrope) and TIME as factors on all the variables described above. The first time point (START) consisted of startLINE, startBEAM, and TRfail, while the second time point (END) included endLINE, endBEAM, and TRsucc. To address our first research question regarding changes during the learning process, we calculated contrasts between TRfail and TRsucc. Furthermore, contrasts between startLINE and endLINE were examined as a baseline to assess the stability of the variable analyzed, as no differences were expected between the two LINE conditions. Contrasts between startBEAM and endBEAM were analyzed to investigate possible transfer effects of learning from one balancing task (tightrope) to another (beam). Contrasts were only conducted if a significant difference was observed in any of the ANOVA outcomes (TASK, TIME, TASK*TIME). To address the second research question, which concerns the differences between less and more common movements, we analyzed the factor TASK and conducted post hoc pairwise comparisons with Bonferroni correction. Prior, sphericity was checked using the Mauchly test (if necessary, Greenhouse–Geisser correction was applied), and normal distribution was verified using the Shapiro–Wilk test. If the normal distribution requirement was violated, an aligned-rank-transformation was performed. This transformation allowed us to conduct factorial ANOVAs on nonparametric data^86–88^ and was implemented using ARTool 2.1.2 software (Washington, USA). Statistical analyses were performed with JASP 0.17.2 (Amsterdam, Netherlands). The alpha level was set at 0.05 and the results were reported at three levels of significance: p < 0.05, p < 0.01, and p < 0.001.

## Results

Participants required 2.6 ± 1.4 attempts (range: 1–5) to perform their first complete gait-cycle with the right leg on the tightrope (TRfail) and 49.4 ± 22.8 attempts (range: 12–101) to complete the learning task (TRsucc). Two participants walked on the tightrope with visual constraints (one participant wore an eye-patch, while the other participant walked with closed eyes). All participants successfully completed the task of walking over the entire line and beam on their first attempts.

### Muscle synergy analyses

#### Overall synergy analyses

**Figure 3:**
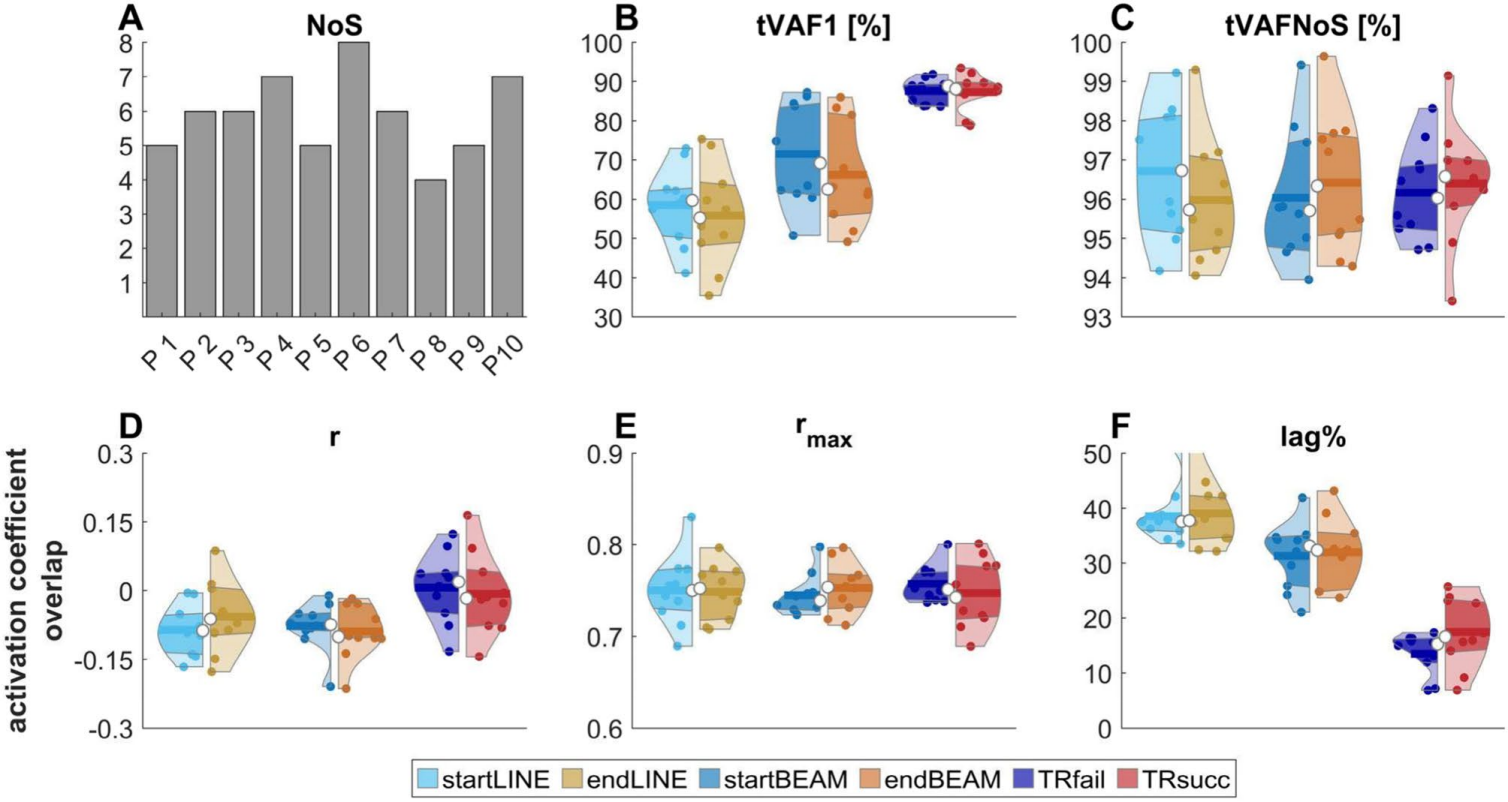
(**A**) Bars show the number of required synergies (NoS—determined by the knee-point of the overall total variance accounted for curve) for each participant (P1–P10) among all conditions; (**B**) the total variance accounted for each condition for one synergy (tVAF1); (**C**) the total variance accounted for each condition at NoS (tVAFNoS). (**D**, **F**) Synergy activation coefficient overlap measured by Pearson correlation (**D**: r), maximum cross-correlation coefficient (**E**: r_max_) and lag at r_max_ (**F**: lag%). Violin plots: each colored circle represents one participant; thick lines represent mean values; white circles indicate median values; dark areas indicate quartiles.

An average of 5.9 ± 1.1 NoS was determined among participants. A significant effect of TASK (p < 0.001) was observed for tVAF1. Post hoc comparisons revealed that tVAF1 was higher in BEAM compared to LINE (p < 0.01), and TIGHTROPE was higher than both LINE and BEAM tasks (p < 0.001). There were no significant differences in tVAFNoS. Regarding the overlap of activation coefficients, the ANOVA revealed a significant effect of TASK for r (p < 0.05), with activation coefficients being more correlated to each other in TIGHTROPE compared to LINE and BEAM tasks (p < 0.05). There was no significant difference for r_max_, but a significant effect of TASK (p < 0.001) and TIME (p < 0.05) for lag%. LINE had a higher lag% than BEAM (p < 0.01), and TIGHTROPE had the lowest lag% (p < 0.001) (Fig. 3).

**Figure 4:**
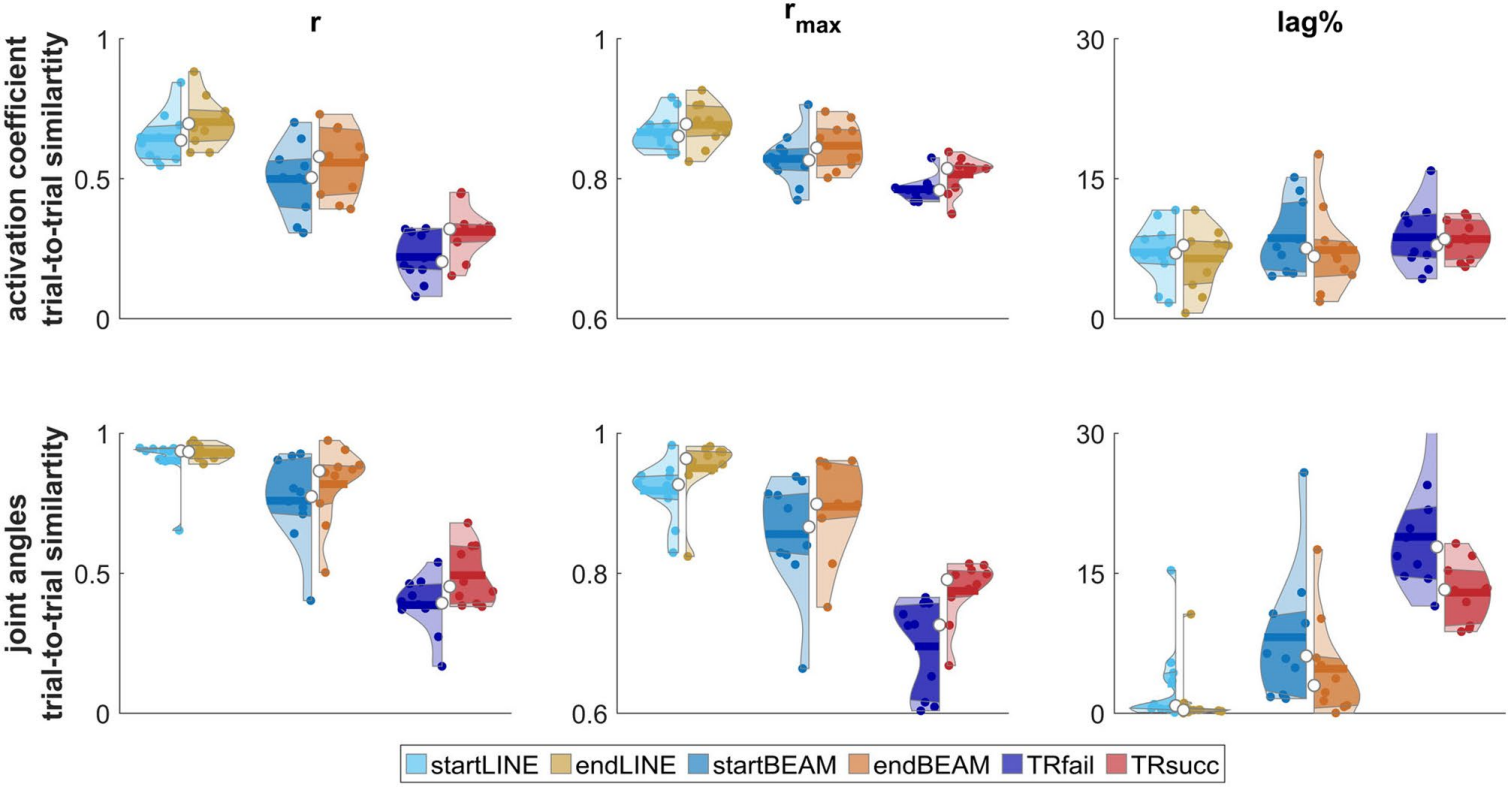
Overall trial-to-trial similarity of synergy activation coefficients (top row) and joint angles (bottom row), measured by Pearson correlation (r), maximum cross-correlation coefficient (r_max_) and lag at r_max_ (lag%). Violin plots: each colored circle represents one participant; thick lines represent mean values; white circles indicate median values; dark areas indicate quartiles.

**Figure 5:**
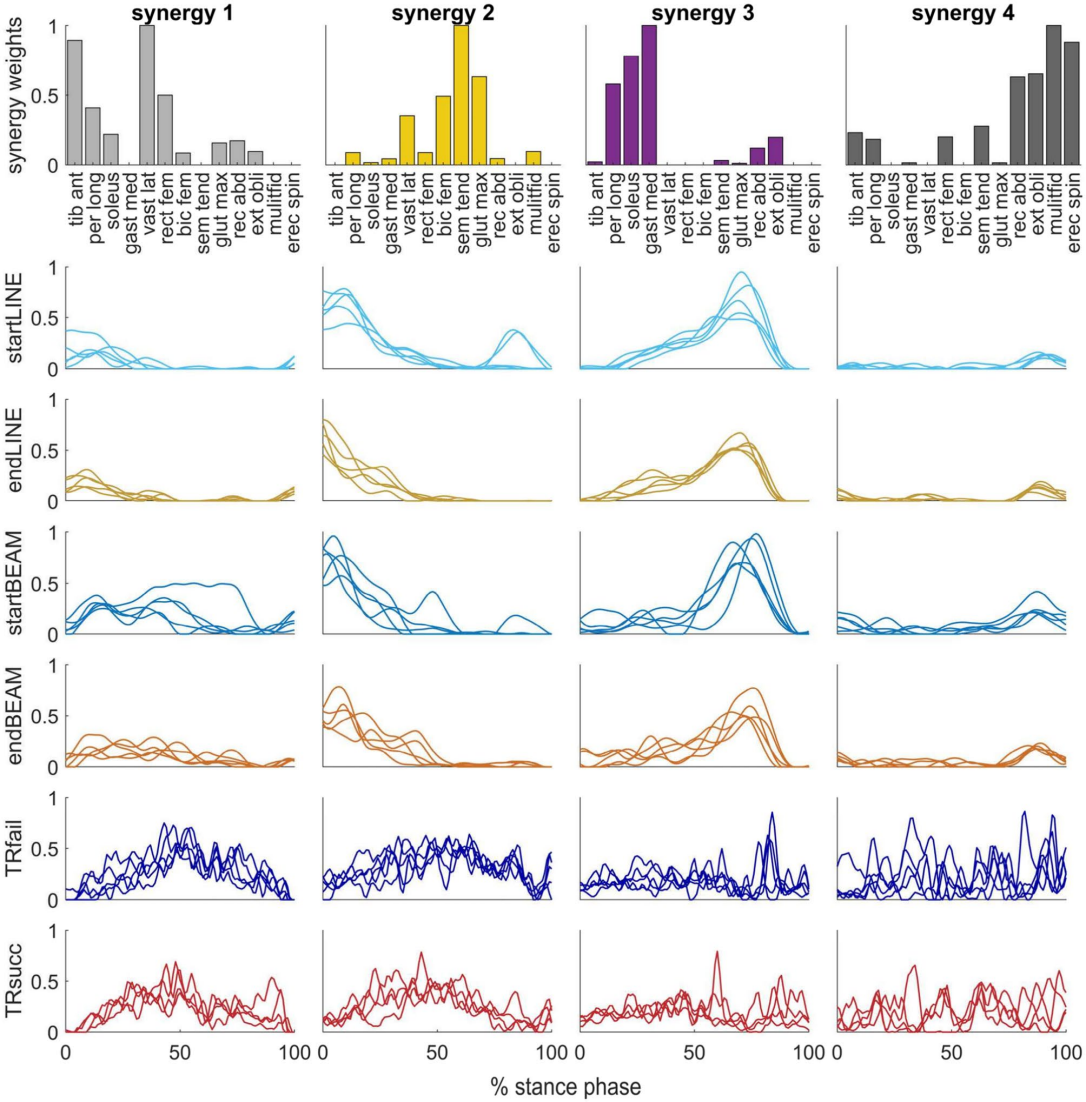
All extracted synergy weights (bar plots) and corresponding activation coefficients (waveform plots in the same column) for each condition of one participant (P8). Each waveform represents the activation coefficient of one trial. Bar colors indicate the cluster, which the synergy weight belongs to, and are the same as in Figs. 6 and 7.

The trial-to-trial similarity, as measured by r and r_max_, was affected by TASK (p < 0.001) and TIME (p < 0.01). The highest values for r and r_max_ were observed in LINE, followed by BEAM (r_max_: p < 0.01; r < 0.001), and the lowest values were observed in TIGHTROPE (both: p < 0.001). Contrasts revealed an increase in similarity from startBEAM to endBEAM (both: p < 0.05) and from TRfail to TRsucc (r_max_: p < 0.05; r: p < 0.001). The ANOVA showed no significant differences for lag% for any of the comparisons (Figs. 4, 5).

#### Individual synergy analyses

**Figure 6:**
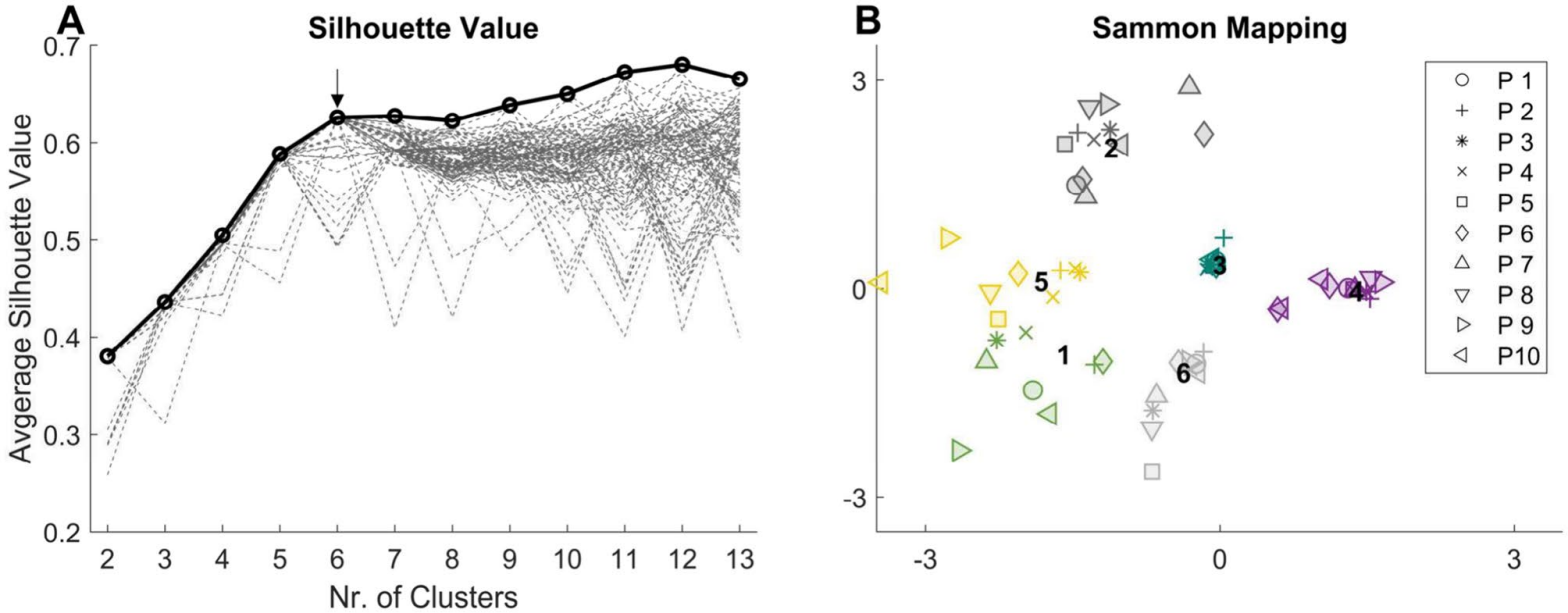
(**A**) Dashed lines show the average silhouette value for each clustering repetition (1–100). The arrow indicates the number of clusters, at which the maximum of averaged silhouette values among repetitions (solid line/circles) plateaued. (**B**) Sammon mapping^104^ of the six clusters. Marker-styles indicate different participants (P1–P10), and marker-colors indicate different clusters. Numbers (1–6) indicate the position of the clusters’ centroids.

**Figure 7:**
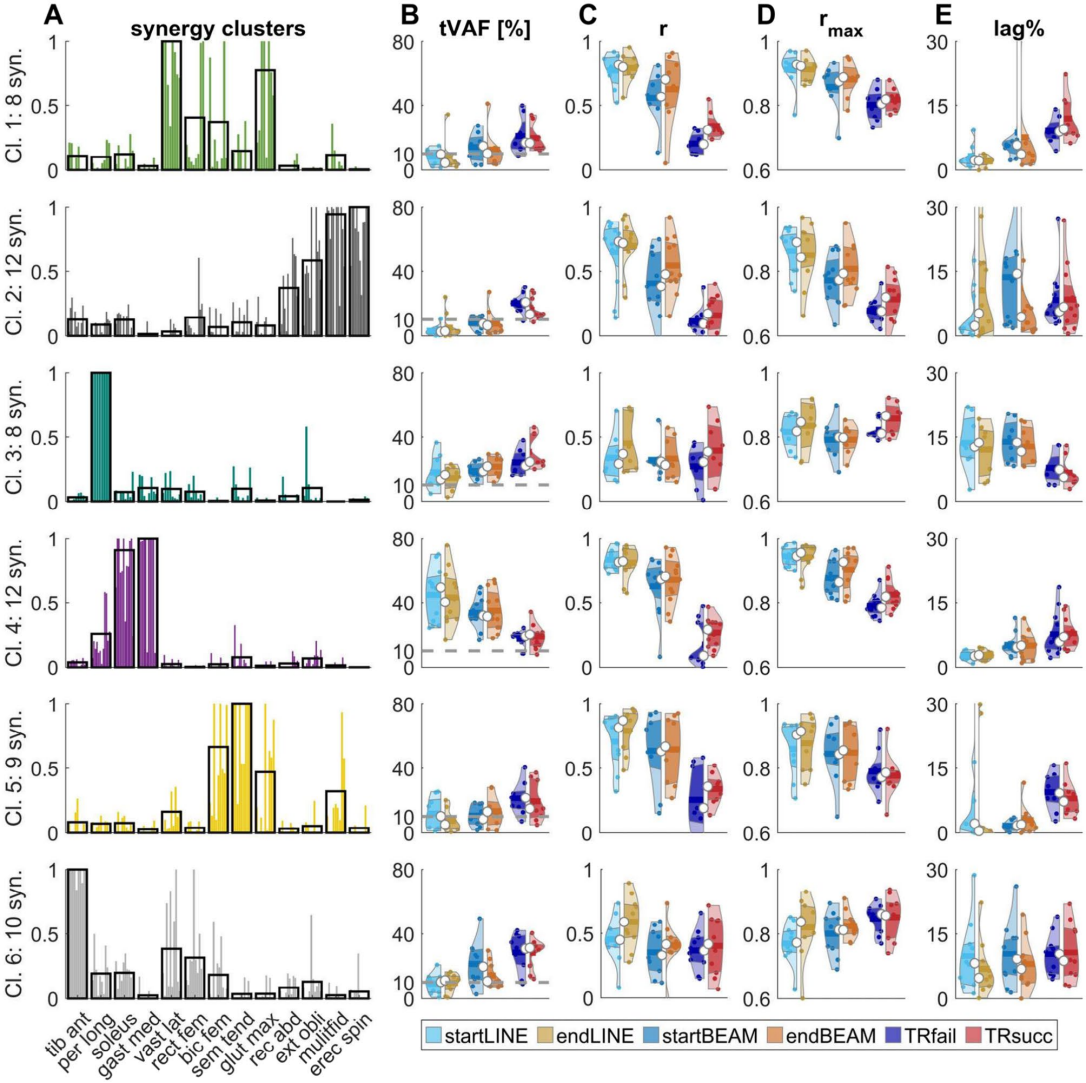
(**A**) Muscle weightings of clustered synergies. Black borders are the cluster (Cl.) centroids, and colored bars (similar to Fig. 6) represent the synergy weights (syn.) that belong to this cluster. (**B–E**) Violin plots represent the total variance accounted for (tVAF), Pearson correlation coefficient (r), cross-correlation coefficient (r_max_) and the lag-time (lag%) for each cluster. Violin plots: each colored circle represents one synergy within the cluster; thick lines represent mean values; white circles indicate median values; dark areas indicate quartiles.

Silhouette analyses identified six clusters (Figs. 6, 7), labelled Cl_1 to Cl_6 in the following paragraphs. Low tVAF values indicate a low contribution of synergy weights to the task. TASK significantly affected the tVAF of all clusters (p < 0.05–p < 0.01). In cluster 4, tVAF of BEAM was lower than LINE (p < 0.05) and the lowest in TIGHTROPE (p < 0.001). For the other clusters, tVAF of TIGHTROPE was higher than BEAM (CL_2, CL_5 and CL_6 p < 0.05–p < 0.001) and LINE (CL_1, CL_2, CL_3, CL_5 and CL_6 p < 0.01–p < 0.001). In BEAM, tVAF was higher than LINE for cluster 2 (p < 0.01). For cluster 2, ANOVA also showed a significant effect of TIME (p < 0.05), with lower tVAF in START compared to END, and the interaction TASK × TIME (p < 0.01). In cluster 6, contrasts revealed a decrease (p < 0.05) of tVAF over time in BEAM.

The trial-to-trial similarity (quantified with r and r_max_) of clusters 1, 2, 4, and 5 was significantly affected by TASK (r_max_: CL_5 p < 0.05; r: CL_5 p < 0.01; others p < 0.001), with higher r (p < 0.001) and r_max_ (CL_5 p < 0.05; others p < 0.001) values for LINE than TIGHTROPE, and higher r values (CL_5 p < 0.01; others p < 0.001) for BEAM than TIGHTROPE. In clusters 1, 2 and 4, r_max_ was higher (p < 0.01–p < 0.001) in BEAM compared to TIGHTROPE. Correlations were higher in LINE than BEAM in clusters 1, 2 and 4 (r and r_max_: p < 0.05–p < 0.001). The lag% showed a significant effect (p < 0.05–p < 0.001) of TASK for clusters 1, 3, 4, and 5. LINE had a lower lag% than BEAM (CL_1 p < 0.05) and TIGHTROPE (CL_1, CL_4 and CL_5 p < 0.01–p < 0.001). BEAM had a lower lag% compared to TIGHTROPE (CL_1 and CL_5 p < 0.01). In contrast, cluster 3 had the lowest lag% in TIGHTROPE compared to the other two tasks (p < 0.05).

Significant effects of TIME were found for r in cluster 1 (p < 0.05), for r_max_ in clusters 4 and 6 (p < 0.05) and for lag% in cluster 4 (p < 0.05), with lower correlations and higher lag% in START compared to END. A significant effect of TASK × TIME was only found for r in cluster 4 (p < 0.05). Contrasts revealed a significant increase of r or r_max_ from startLINE to endLINE in cluster 6 (r_max_: p < 0.05), from startBEAM to endBEAM in cluster 2 (r: p < 0.01), and from TRfail to TRsucc in cluster 1 (r: p < 0.05).

### Kinematic analysis

**Figure 8:**
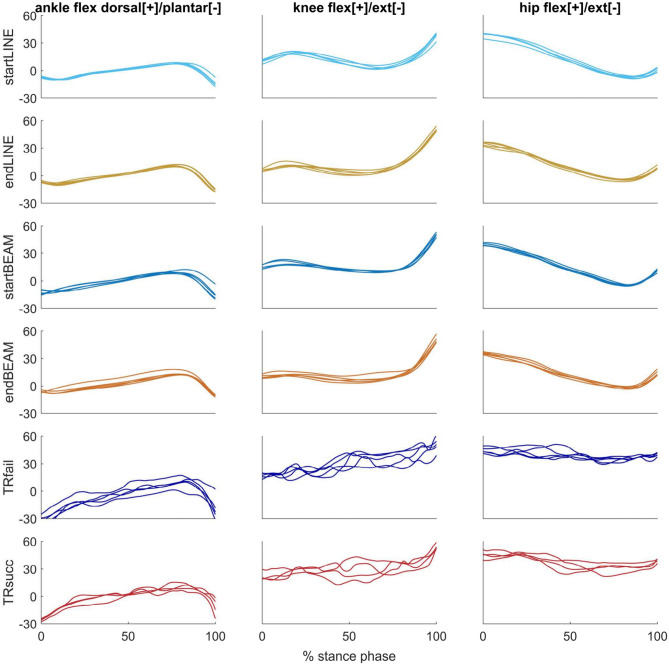
Example of joint kinematic waveforms from one participant (P8). Each line represents one trial per condition. *flex* flexion, *ext* extension.

The overall trial-to-trial similarity of joint angles, quantified by r, r_max_ and lag%, was significantly affected by TASK (p < 0.001). LINE had the highest correlations and the lowest lag% between trials, followed by BEAM, and TIGHTROPE (r_max_ LINE vs BEAM: p < 0.01; lag% LINE vs BEAM: p < 0.05; others: p < 0.001). There was a significant effect of TIME on r (p < 0.05), with lower r in START compared to END, and a significant interaction effect of TASK × TIME (p < 0.05). For r_max_, TIME had a significant effect (p < 0.01), with an increase observed between START and END. All contrasts were significantly different (p < 0.05). Likewise, lag% was significantly affected by TIME (p < 0.01). Contrasts revealed higher lag% (p < 0.05) in startLINE and TRfail compared to endLINE and TRsucc, respectively (Figs. 4, 8).

## Discussion

The study aimed to enhance our understanding of motor learning through synergy analysis, employing a within-participant, within-session study design. We observed reduced overlap and increased trial-to-trial similarity of activation coefficients after learning and with increasing movement proficiency. Additionally, our analyses revealed that the contribution of specific synergy weights to movements vary across tasks.

Over half a century ago, Bernstein^5^ proposed that in the early stages of learning, the number of possible movement executions is minimized to the essential elements required to master the fundamental aspects of a skill, thereby simplifying control. This concept, called “freezing the degrees of freedom”, has primarily been studied at the kinematic level. Guimarães et al.^89^ conducted a review focusing on joint kinematics and showed that during the initial learning phase, the joint range of motion is reduced and there is a higher temporal coupling of joint angles (e.g., adjacent joints extend/flex simultaneously) compared to a state of sufficient proficiency. However, to our knowledge, the mechanisms by which the central nervous system simplifies motor control in early learning remain unclear. Steele et al.^74^ found greater overlap of synergy activation coefficients when biomechanical and task constraints were present. Our study showed higher tVAF1 and greater overlapping of synergy activation coefficients—both indicating higher co-activation of synergies—in movements with low (i.e., walking over a tightrope) compared to movements with high (i.e., walking on a line) proficiency. In light of these findings, we hypothesize that in movements with low proficiency and during early learning, the temporal co-activation of synergies represents a simplification strategy of the central nervous system, which leads to the freezing of the degrees of freedom. While this hypothesis calls for further research, we are confident that a high temporal overlap of activation coefficients represents a compensation strategy of the central nervous system to promote robust motor control. Similarly, previous studies have demonstrated that activation coefficients were activated over a longer time period during and following gait perturbations compared with unperturbed walking^90–92^, and in patients with neurological disorders compared to healthy individuals^93–95^. The authors concluded that the longer recruitment of activation coefficients, corresponding to a greater temporal overlap, was a strategy to account for the higher demands during challenging locomotion tasks. Our study supports this theory and implies a modulation of the temporal overlap due to proficiency and during early learning.

Motor complexity has been assessed previously by the tVAF and NoS^16–18,20,21^. However, high tVAF values may arise from overlapping activation coefficients, representing a simplification strategy of the central nervous system to minimize the number of possible movement solutions, rather than a simpler motor control due to a decreased number of synergies necessary for the task per se. This idea is supported by previous studies on both impaired and unimpaired populations. Clark et al.^21^ found similar synergy weights in locomotion for stroke survivors and unimpaired individuals, when the same number of synergies were extracted, rather than the number determined by a tVAF threshold. The authors concluded that the spatial synergy weights did not differ, but that they were computationally merged by the factorization algorithm due to their overlapping recruitment profiles. Similarly, merging of synergy weights has been observed in post-stroke survivors^96^, individuals with Parkinson’s disease^42^ and people after cortical lesions^97^. In summary, we propose that one simplification strategy of the central nervous system to promote robust motor control is to co-activate muscle synergies. This can be measured by the tVAF and the temporal overlap of synergy activation coefficients.

Our findings indicate that the temporal overlap of activation coefficients can have a big impact on muscle synergy analyses, including the number of shared synergies. Factorization algorithms extract synergies based on muscle co-activation. Therefore, computational merging of synergies due to overlapping activation coefficients may result in synergies that do not accurately reflect the underlying mechanisms in the central nervous system. Previous studies on real and simulated datasets^69,73,74^ have observed this phenomenon. In these studies, increased temporal overlap of activation coefficients led to merging of synergy weights due to the underlying assumptions of the factorization algorithms. In the current study, we found a small number of shared synergies when calculated separately for each condition (see Supplementary material), but similar synergies when calculated over all conditions. Additionally, our results showed a great temporal overlap of activation coefficients in the low compared to the high proficiency movement tasks. These findings suggest that similar synergies were used for the tasks, but high overlap of activation coefficients during the low proficiency tasks may have resulted in the low number of shared synergies. In previous studies, a lower amount of shared synergies between overground walking and balancing tasks has been found in individuals with no dance experience compared to expert dancers^8,31^, in stroke survivors compared to unimpaired individuals^98^, and prior to a dance-based rehabilitation in individuals with Parkinson’s disease^32^. Two of these studies also found lower spatial distinctness of synergy weights in individuals with no dance experience^31^, and before the dance-based rehabilitation^32^. The lower distinctness of the computed synergy weights may be a result of a greater overlap of activation coefficients, which can compromise the extracted synergy weights. Consequently, merged synergy weights could be a reason for the lower number of shared synergies in previous studies. Additionally, Santuz et al.^62^ demonstrated that the amount of merged synergy weights could vary depending on the factorization algorithm employed and called for greater awareness in attributing a neurophysiological nature to highly merged synergies. These findings emphasize the need for caution when interpreting muscle synergy results, especially in relation to shared synergies, tVAF and NoS. To reduce the impact of computational merging, we calculated synergies over the entire dataset for each participant rather than extracting each condition separately. This approach holds potential for advancing synergy research and is discussed in more detail in the Supplementary material.

The current study showed an increase in trial-to-trial similarity of activation coefficients during learning and with higher movement proficiency (Fig. 4). These results support previous research on trial-to-trial similarity as outlined in the introduction^23–26,28,30,31^. In terms of the overall trial-to-trial similarity of activation coefficients, we observed a transfer effect of a balance training from the tightrope to the beam task. However, there were no differences between startLINE and endLINE, suggesting that differences did not occur due to movement-artefacts or sensor-noise. Through cluster analysis, we were able to detect changes in similarity in certain synergies (Fig. 7). Cluster 6—mainly formed by tib_ant, vast_lat and rec_fem—did not show any changes in similarity due to proficiency or learning. Additionally, cluster 3—mainly formed by per_long—had a lower lag% in tightrope walking compared to the other two walking tasks. We speculate that the recruitment of synergies belonging to these two clusters is not crucial for different levels of proficiency or learning. The other clusters showed that trial-to-trial similarity increases with movement proficiency. While we observed an increase of trial-to-trial similarity from startBEAM to endBEAM and from TRfail to TRsucc in certain clusters, other clusters showed no changes throughout the learning process. This suggests that early learning is driven by an increase in the consistency of certain synergies, while other synergies increase their consistency in a later learning stage, i.e., at higher proficiency levels.

The duration of analyzed movements can affect muscle synergy analyses due to differences in smoothing of EMG envelopes^36^. In our study, stance phase duration differed between tasks and between TRsucc and TRfail. Specifically, stance phases were shorter in TRsucc than TRfail (Supplementary material). This explains the smoother synergy activation coefficients in LINE and BEAM tasks compared to the TIGHTROPE task^36^ (Fig. 5). One could assume less trial-to-trail similarity in TIGHTROPE compared to the other two tasks due to less smoothed activation coefficients. However, this would not explain the differences in similarity between LINE and BEAM tasks, as stance duration did not differ between these two tasks. To further evaluate whether our findings were affected by the different task durations, we adjusted the low-pass cutoff filter frequency for each trial based on its duration and repeated our main analyses on synergies. Detailed information and results for these additional analyses are provided in the Supplementary material. Briefly, these analyses showed similar results in terms of trial-to-trial similarity and overlap of activation coefficient between the tasks. Moreover, the temporal overlap of activation coefficients decreased from TRfail to TRsucc. In summary, the additional and main analyses yielded the same conclusions. Namely, fine tuning of synergy recruitment, i.e. increasing trial-to-trial similarity and decreasing temporal overlap of activation coefficients, is important for motor learning. This viewpoint is corroborated by studies conducted by Berger et al.^38–40^, which demonstrated the significance of activation coefficient adaptation in early motor learning. We hypothesize that upon a completion of the learning process, indicated by the successful completion of all attempts at TIGHTROPE walking (rather than just 4 out of 5), the trial-to-trial similarity and the overlap of activation coefficients will be comparable to those observed in LINE and BEAM tasks.

To gain a more comprehensive understanding of changes in trial-to-trial similarity, we also examined changes in similarity of joint angles (Figs. 4, 8). Surprisingly, the overall trial-to-trial similarity of kinematic data was not only higher with proficiency and after learning, but also in endLINE compared to startLINE. Therefore, we hypothesize that synergies reflect motor planning by the central nervous system, whereas joint kinematics is more influenced by peripheral noise in the movement execution^22,23^. Further analysis of the trial-to-trial similarity of EMG envelopes yielded comparable results to those observed in the overall synergy analyses with regard to task proficiency (see Supplementary material).

In the field of motor learning and development, three theories are widely discussed: the strict nativist view, the learning hypothesis, and the combined approach^28^. The strict nativist view proposes that locomotor patterns remain robustly conserved into adulthood, supported by the spatial synergy model^42,58^ and studies analyzing stepping patterns in newborns^99^. The learning hypothesis proposes that unstructured movement patterns are transformed into structured solutions during development through the interaction between the body and the environment, supported by studies showing low trial-to-trial similarity of both kinematics and synergies in early learning^23–26,28,30,31^. The combined approach posits the existence of conserved movement patterns that are enriched with new patterns to represent a wider range of tasks. The latter concept has recently been supported by analyses of muscle synergies in the development of walking^28^ and running^100^. In our study, we found similar synergy weights across tasks (see Supplementary material for a more detailed discussion). Besides similar synergy weights, we observed lower consistency in activation coefficients at low compared to high proficiency levels. Moreover, some synergy weights showed a minimal contribution to the line and beam tasks but were important for tightrope walking (Fig. 7), hinting towards an enrichment of the motor control repertoire. These findings therefore provide support for the combined theory for motor learning.

Our study included three notable limitations. Firstly, due to the intra-session design, we captured a limited number of gait cycles per condition. Oliveira et al.^41^ suggested extracting muscle synergies over a minimum of 20 concatenated steps to account for trial-to-trial variability in movement execution. To address this, we performed our main analysis on concatenated data of all conditions, including 24–30 stance phases per participant. Secondly, we defined the learning process as complete when participants successfully walked across the entire tightrope in four out of five consecutive attempts, which may not reflect a high level of proficiency. Nonetheless, despite this limitation, we observed significant changes in most of the parameters analyzed between TRfail and TRsucc. Thirdly, the study population was relatively small, consisting of ten young recreational athletes. Although this sample size aligns with most synergy studies^10,30,32,78^, future research should include a larger and more diverse participant pool. The study findings should be interpreted with consideration to the potential differences in learning rate and process between different populations, such as young and elderly individuals^30,101–103^.

In summary, we found that increasing movement proficiency leads to less overlap and higher trial-to-trial similarity of synergy activation coefficients. We propose that the higher temporal overlap represents a strategy employed by the central nervous system to promote robust motor control in challenging tasks with low proficiency and early learning. Furthermore, we suggest that fine-tuning of synergy activation coefficients is essential for motor learning.

### Supplementary Information

Supplementary Information.

## Data Availability

The raw and processed EMG signals, joint angles, and events (start and end of stance phases) are available as anonymized data on https://osf.io/x52je/?view_only=7f051720558a421e957a316bad1c0992. For c3d files and Octave codes, contact Paul Kaufmann at paul.kaufmann@univie.ac.at or Hans Kainz at hans.kainz@univie.ac.at. The authors will assist with any reasonable replication attempts for 2 years following publication.
