# Supplementary material for: Increased trial-to-trial similarity and reduced temporal overlap of muscle synergy activation coefficients manifest during learning and with increasing movement proficiency
Source: Sci Rep. 2024 Jul 31;14:17638. doi: 10.1038/s41598-024-68515-3 (PMC11291506; doi:10.1038/s41598-024-68515-3)
Supplement: Supplementary file 1 — Supplementary Information. [file 41598_2024_68515_MOESM1_ESM.docx]

Supplementary Material

**Increased trial-to-trial similarity and reduced temporal overlap of muscle synergy activation coefficients manifest during learning and with increasing movement proficiency**

Running Title: **muscle synergies during motor learning**

Paul Kaufmann^1,2^, Willi Koller^1,2^, Elias Wallnöfer^1,2^, Basilio Goncalves^1,2^, Arnold Baca^1^, Hans Kainz^1,2*^

^1^Department of Biomechanics, Kinesiology and Computer Science in Sport, Centre for Sport Science and University Sports, University of Vienna, Vienna, Austria

^2^Neuromechanics Research Group, University of Vienna, Vienna, Austria

**^*^Correspondence:**Ass-Prof Hans Kainz, PhD; Head of the Neuromechanics Research Group; <https://neuromechanics.univie.ac.at/>; [hans.kainz@univie.ac.at](mailto:hans.kainz@univie.ac.at); +43 1 4277 48887 Austria, 1150 Vienna, Auf der Schmelz 6a, ORCID: 0000-0001-5296-6040

**Contributing authors:**

Paul Kaufmann, MSc; [paul.kaufmann@univie.ac.at](mailto:paul.kaufmann@univie.ac.at); Austria, 1150 Vienna, Auf der Schmelz 6a, ORCID: [0000-0002-3095-3836](https://orcid.org/0000-0002-3095-3836)

Willi Koller, MSc; [willi.koller@univie.ac.at](mailto:willi.koller@univie.ac.at); Austria, 1150 Vienna, Auf der Schmelz 6a, ORCID: 0000-0001-7562-0624

Elias Wallnöfer, MSc; [elias.kaj.wallnoefer@univie.ac.at](mailto:elias.kaj.wallnoefer@univie.ac.at); Austria, 1150 Vienna, Auf der Schmelz 6a, ORCID: 0009-0007-9195-3323

Basilio Goncalves, PhD; [basilio.goncalves@univie.ac.at](mailto:basilio.goncalves@univie.ac.at); Austria, 1150 Vienna, Auf der Schmelz 6a, ORCID: 0000-0002-2757-2979

Prof Arnold Baca, PhD; [arnold.baca@univie.ac.at](mailto:arnold.baca@univie.ac.at); Austria, 1150 Vienna, Auf der Schmelz 6a, ORCID: 0000-0002-1704-0290

Ass-Prof Hans Kainz, PhD; [hans.kainz@univie.ac.at](mailto:hans.kainz@univie.ac.at); Austria, 1150 Vienna, Auf der Schmelz 6a, ORCID: 0000-0001-5296-6040

# Synergy extraction – spatial synergy model

As stated in the main paper, the spatial synergy model describes EMG signals as a linear combination of fixed synergy weights and time dependent activation coefficients [1-3]. Equation (1) presents the mathematical concept behind this, where E is the EMG matrix, C is the activation coefficient, W is the synergy weight and e is the residual error. The subscript *mus* indicates the number of muscles (here 13) and *tps* the number of timepoints (here the number of all trials per participant multiplied by 101). Note that *k* represents the number of synergies extracted and ranges from 1 to 12 (mus -1) in the current study, while *g* represents the synergy number (1 to *k*). The non negative-matrix-factorization (NNMF) algorithm [4-6] aims to obtain the smallest possible residual error in this equation by updating C and W over numerous iterations.

$${\left( 1 \right) E}_{mus \times tps}= \sum_{g=1}^{k} {C(g)}_{k\times tps} {W(g)}_{mus\times k}+e$$

We used an advanced NNMF algorithm introduced by Kim & Park [7] based on the block principal pivoting method for the non-negativity constrained least squares problem. In this method, convergence is achieved as a stopping criterion, in contrast to the classical NNMF method, which could get stuck in local minima. The “nmf_bpas” octave function was used with 50 to 5000 allowed iterations to obtain a convergence criterion of 10^-5^ for f in equation (2). Both α and β represent the mean of E (but one could give each an individual initial guess instead), and the subscript F indicates the Frobenius norm.

$$\left( 2 \right) f\left( W, C \right)= \frac{1}{2}(\left\| E-WC \right\|_{F}^{2}+\alpha\left\| W \right\|_{F}^{2}+\beta\left\| C \right\|_{F}^{2}$$

Research has demonstrated that the number of iterations required for a NNMF algorithm to converge and its final solution is strongly influenced by the initialization (= first guess) of W and C, which are - if not specified - random inputs [8-10]. Recently, in muscle synergy analyses, initialization methods such as single-value-decompensation (SVD), principal-component-analysis, or spatial distributions have gained attention due to their positive effects on hastening the NNMF algorithm and performing better when activation coefficients of the different synergies are more correlated [2, 11]. Therefore, we here used the NNSVDLRC (nonnegative single-value-decompensation with low-rank correction) function, introduced by Atif et al. [9], with default inputs (stop criterion: 0.05; maximum number of iterations: 20) to obtain better initial guesses for W and C. This algorithm was designed to improve performance at low ranks (k), which are important in muscle synergy analysis.

# k-means clustering

As stated in the main paper, we utilized octave’s built-in “kmeans” function to cluster similar synergies among participants. The following properties were applied: squared Euclidean distance; k-means++ initialization algorithm; maximum iteration number of 10^100^ to achieve a change in any centroid less than 0.0001; 5000 replicates.

# Task duration

## Methods

Previous studies found shorter gait-cycle durations after locomotor development [12-14]. To evaluate whether stance-phase durations were shorter with higher proficiency and after a learning process, the durations of all trials within one condition were averaged and compared across conditions using a 2-way ANOVA (see main paper statistics).

## Results

A significant effect of TASK (p < 0.001), TIME (p < 0.05) and the interaction TASK × TIME (p < 0.05) was found on the duration of stance phases (Supplementary Figure S1). TIGHTROPE had longer stance phases (p < 0.001) than LINE and BEAM, with no difference between the latter two. Contrasts showed significantly shorter stance phases in TRsucc than Trfail (p < 0.05; Supplementary Figure S1).


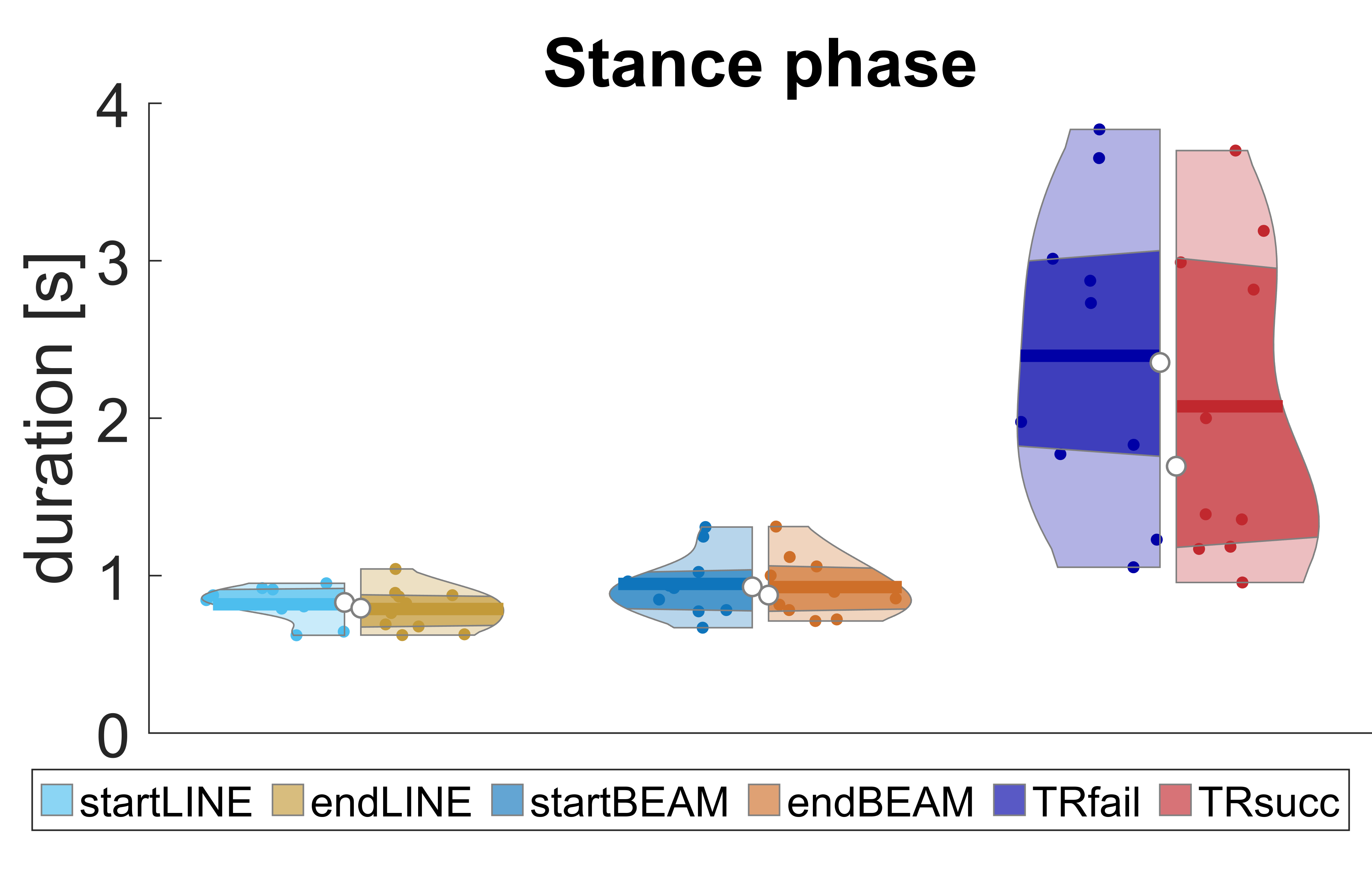
 Supplementary Figure S1: Stance phase duration (in seconds [s]) of each condition. Violin plots: each colored circle represents one participant; thick lines represent mean values; white circles indicate median values; dark areas indicate quartiles.

# EMG – trial specific low pass cutoff frequency

## Methods

Reviews by Hug et al. [15] and Turpin et al. [2] call for greater awareness of the choice of cutoff frequencies for low-pass filters prior to synergy extraction. In simple terms, a lower cutoff frequency results in a smoother EMG envelope. This ‘wider’ activation profile contains less variation and probably more overlap between different muscle profiles, which naturally affects synergy results. Several studies have investigated the effect of different low-pass filters on extracted synergies. For instance, lower cutoff frequencies led to a higher total variance accounted for (tVAF) at a given number of synergies, which consequently also affected the choice of the number of required synergies (NoS) for a movement[15-19]. However, Hug et al. [16] demonstrated that NoS was not affected by different cutoff frequencies when the knee-point method was applied (as also used in the current study), in contrast to fixed thresholds (e.g. tVAF ≥ 90%). Furthermore, the use of low-pass filters has been found to affect both synergy weights and activation coefficients extracted via NNMF [18, 19]. This presents a problem not only when comparing various studies but also when investigating movements with different durations within the same study [15]. To address this issue, recent studies have attempted to achieve similar smoothed electromyography (EMG) profiles by determining the low-pass cutoff frequency relative to movement duration, e.g. 5 – 12 Hz for 60 – 140% of an optimal pedaling rate according to pedaling rates [20], 9 Hz for walking and 12 Hz for pedaling based on a machine learning pattern recognition algorithm [21], or by dividing a fixed cutoff frequency by the trial specific duration, i.e. 3.5 Hz/duration for treadmill walking [22] and 7 Hz/duration for overground walking of post-stroke patients [23]. In the current study, we used the same procedure as Banks et al. [23] and set a trial specific cutoff frequency for the low-pass filter - 7 Hz/stance-phase-duration. All other EMG processing and synergy extraction steps were performed in the same manner as our main analysis (main paper). We aimed to determine whether the different durations would alter our main synergy results in terms of complexity, overlap and trial-to-trial similarity. Supplementary Figure S2 illustrates how different cutoff frequencies affect EMG smoothing.


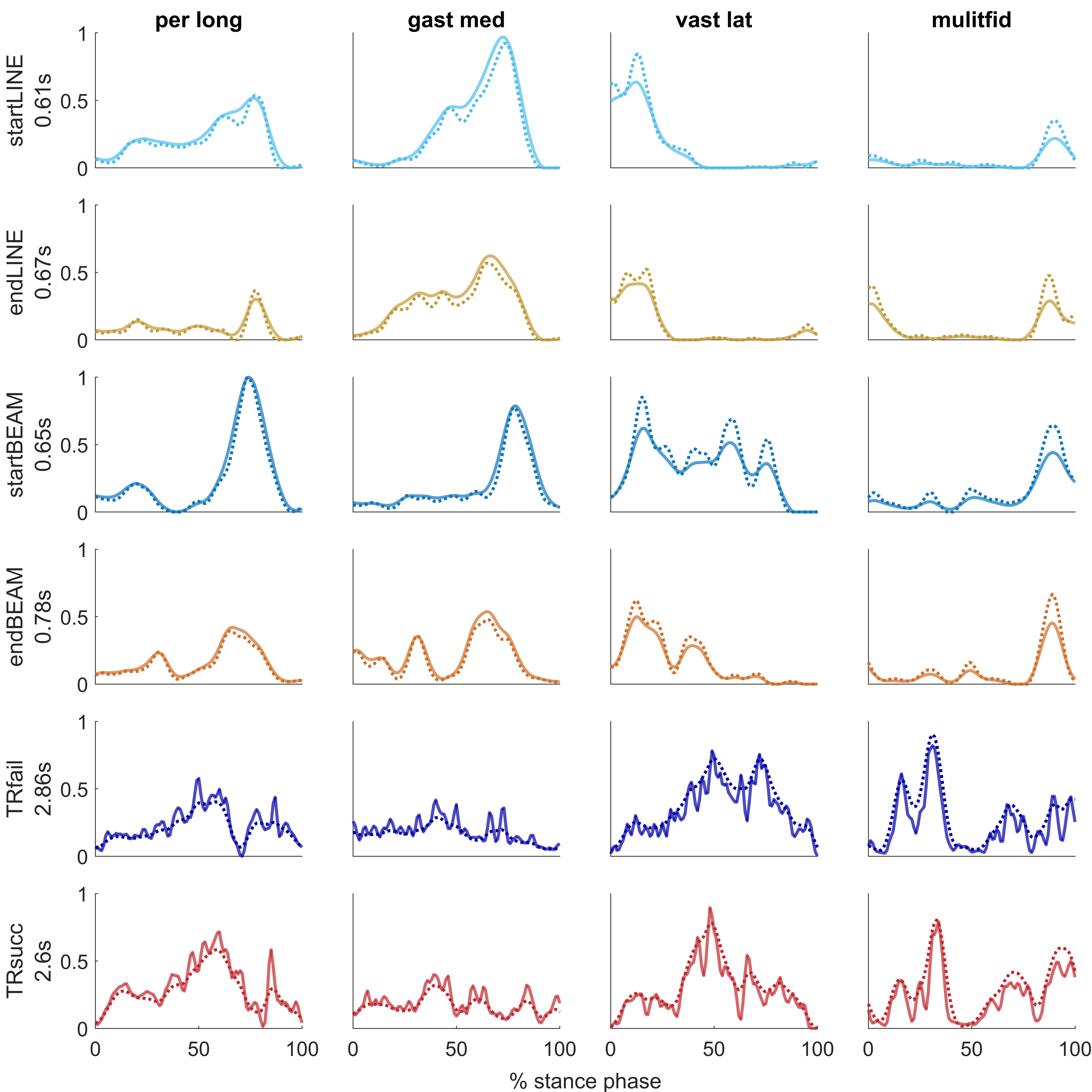


Supplementary Figure S2: Example of the influence of different cutoff frequencies on EMG smoothing of 4 muscles (1 trial per condition, of 1 participant). The solid lines represent the fixed low-pass cutoff frequency (7 Hz), and the dashed lines represent the duration-dependent cutoff frequency (7 Hz/trial duration). The duration is shown on the y axis and given in seconds [s].

## Results

An average of 5.6 ± 2.22 NoS was determined among participants. For tVAF1 a significant effect of TASK (p < 0.001) was observed, with highest tVAF1 in TIGHTROPE, followed by BEAM and LINE at last (p < 0.001). There was also a significant effect of TASK in tVAFNoS (p < 0.05) which was higher in TIGHTROPE compared to LINE (p < 0.05). Regarding the overlap of activation coefficients, the ANOVA revealed a significant effect of TASK for r (p < 0.02), r_max_ and %lag (p < 0.001). Activation coefficients were more correlated to each other (r: p < 0.01; r_max_: p < 0.001) in TIGHTROPE compared to LINE and BEAM. The lag% was higher in LINE than BEAM (p < 0.05) and lowest in TIGHTROPE (p < 0.001). Additionally, r_max_ was significantly affected by the interaction TASK × TIME (p < 0.05), where contrasts revealed a decrease during learning on the TIGHTROPE (p < 0.05) (Supplementary Figure S3).

Regarding trial-to-trial similarity, there was a significant effect of TASK (p < 0.001) on r, with highest correlations in LINE, followed by BEAM (p < 0.01) and TIGHTROPE at last (p < 0.001). Correlation was significantly affected by TIME (p < 0.05). Contrasts revealed an increase during learning on the TIGHTROPE (p < 0.05). There were no significant differences in cross-correlations r_max_ and %lag (Supplementary Figure S3, Supplementary Figure S4).


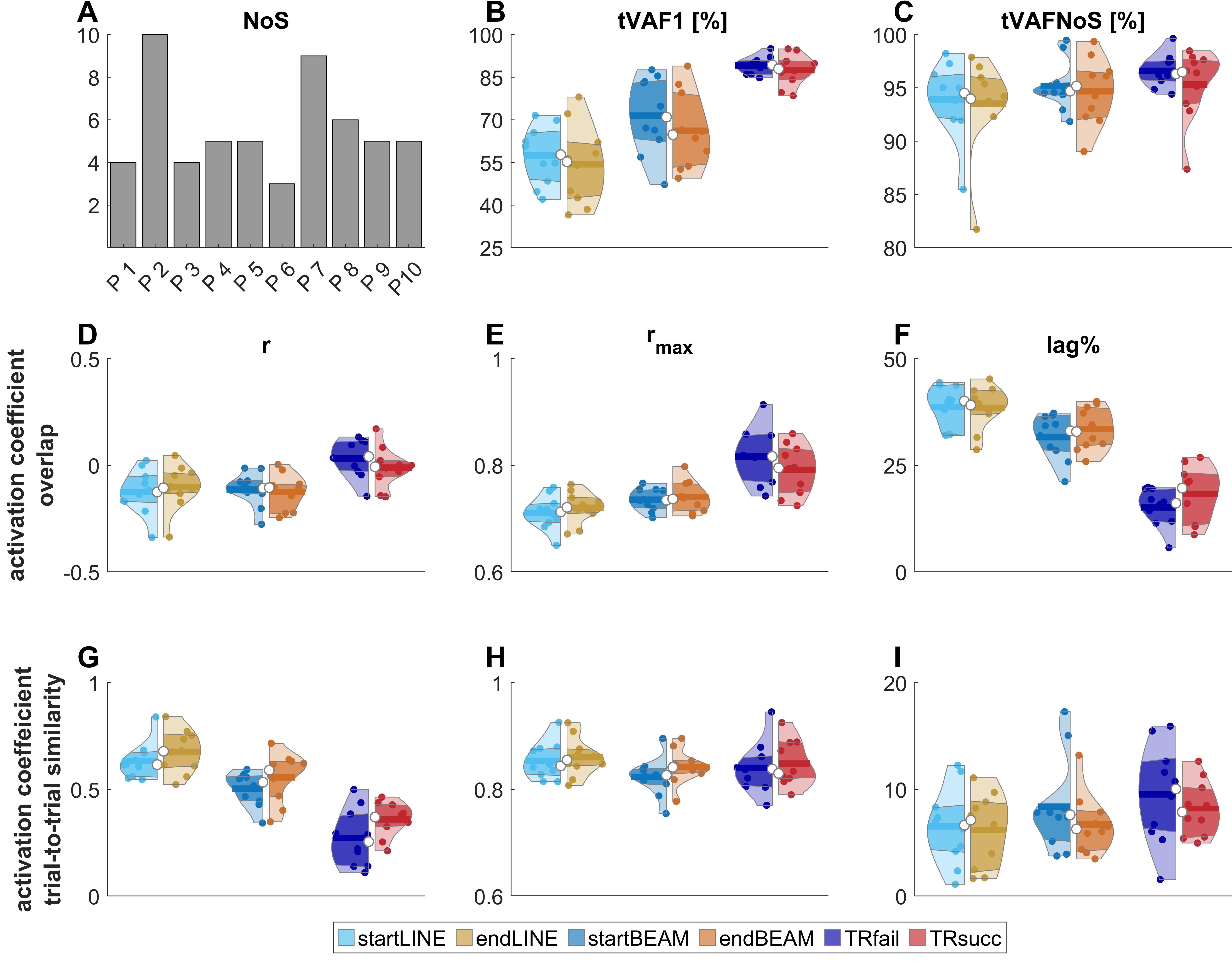


Supplementary Figure S3: Synergies were extracted of EMG signals filtered with the duration-dependent cutoff frequencies. A: bars show the number of required synergies (NoS) for each participant (P1 – P10). B-C: the total variance accounted for one synergy (B: tVAF1) and NoS (C: tVAFNoS). D-F: Synergy activation coefficient overlap and G-I: trial-to-trial similarity measured by Pearson correlation (D, G: r), maximum cross-correlation coefficient (E, H: r_max_) and lag at r_max_ (F, I: lag%). Violin plots: each colored circle represents one participant; thick lines represent mean values; white circles indicate median values; dark areas indicate quartiles.


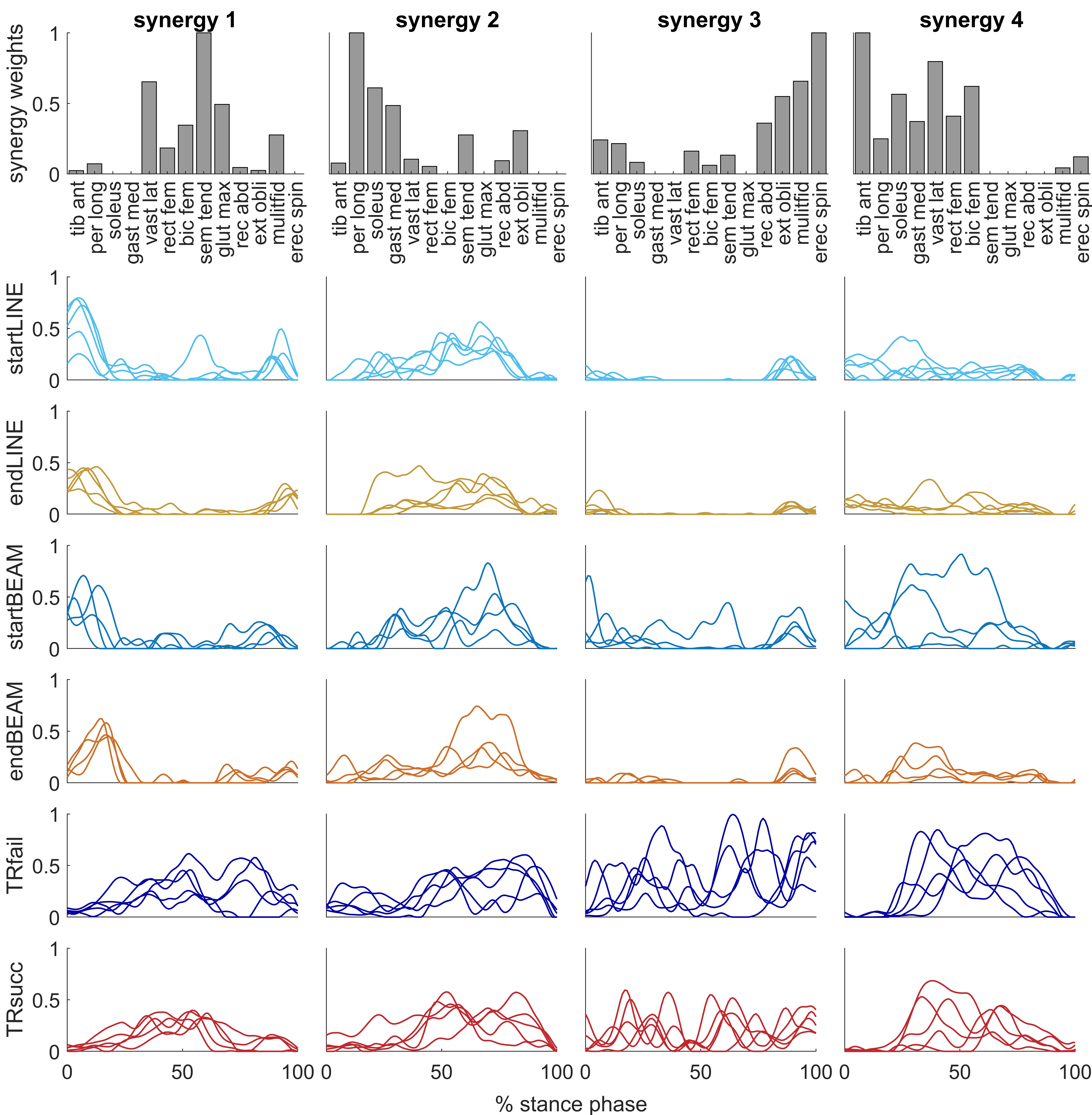


Supplementary Figure S4: All extracted synergy weights (bar plots) and corresponding activation coefficients (waveform plots in the same column) for each condition of one participant (P3). Each waveform represents the activation coefficient of one trial.

# Muscle synergy extraction from each condition independently

## Methods

To investigate whether similar synergy weights were used across different walking conditions, we performed individual synergy extraction for each condition using the procedures described in the main paper. To do this, we concatenated the EMG matrices within each condition and used NNSVDLRC and NNMF. We then performed two widely used analyses on this data. In the first step of our analysis, we compared the synergy weights across all possible pairs of conditions using the Pearson correlation coefficient (r). A pair of synergy weights was considered as similar (= shared), if r > 0.684, which corresponds to the critical value of r for 13 muscles at p = 0.01 [24-30]. To account for variations in the number of synergies (NoS) across conditions and participants, we visualized the number of shared synergies (n_shared_) as percentage. Equation (3) was used to calculate the percentage of shared synergies (%n_shared_) between two conditions, where subscripts *condition1* and *condition2* indicate the two compared conditions (e.g., startLINE and endLINE).

$$\left( 3 \right) \%n_{shared}=100 \frac{n_{shared}}{{min (NoS}_{condition1}, {NoS}_{condition2})}$$

In the second step, we reconstructed the activation coefficients for all conditions using the synergy weights from either startLINE or startBEAM. We employed a commonly used reconstruction algorithm [20, 29, 31-35] based on the updating rule for NNMF proposed by Lee and Seung [5], as described in equation (4). Synergy weights (W) from one condition were held fixed (suffix: fix) to reconstruct activation coefficients (C) (suffix: rec) with the corresponding EMG matrix (E) from another condition. After an initial random guess for the reconstruction matrix, numerous iterations (n, here ranging from 50 to 5000) were performed until the function f(W,C) reached a convergence criterion (10^-5^) described in equation (5). Subscripts i and j indicate the row and column, while superscript T indicates the transposed matrix. Crec was used to calculate the reconstructed tVAF (tVAFrec), together with E and Wfix. Consistent with previous studies [35-37], synergy weights were assumed to be similar across conditions when tVAFrec exceeded 80%.

$$\left( 4 \right) {Crec}_{ij}^{(n)}={Crec}_{ij}^{(n-1)}\left( \frac{{({Wfix}^{T} E)}_{ij}}{{({Wfix}^{T} Wfix {Crec}^{(n-1)})}_{ij}} \right);$$

$$\left( 5 \right) f\left( Wfix, Crec \right)=\frac{\left\| E-Wfix Crec \right\|_{F}}{\sqrt{m n}}$$

## Results

Supplementary Figure S5 shows the high amount of shared synergy weights across the LINE and BEAM tasks for all participants. A high range, and on average a smaller %n_shared_ was found between LINE or BEAM conditions with TIGHTROPE conditions across participants. Reconstruction procedures revealed that tVAFrec was > 80% in all participants for all LINE and BEAM conditions. In contrast, only six participants exceeded the threshold in TIGHTROPE conditions when activation coefficients were reconstructed using synergy weights of startLINE. When activation coefficients were reconstructed using startBEAM, tVAFrec of one participant was below our criterion for TRfail and two participants for TRsucc (Supplementary Figure S5).


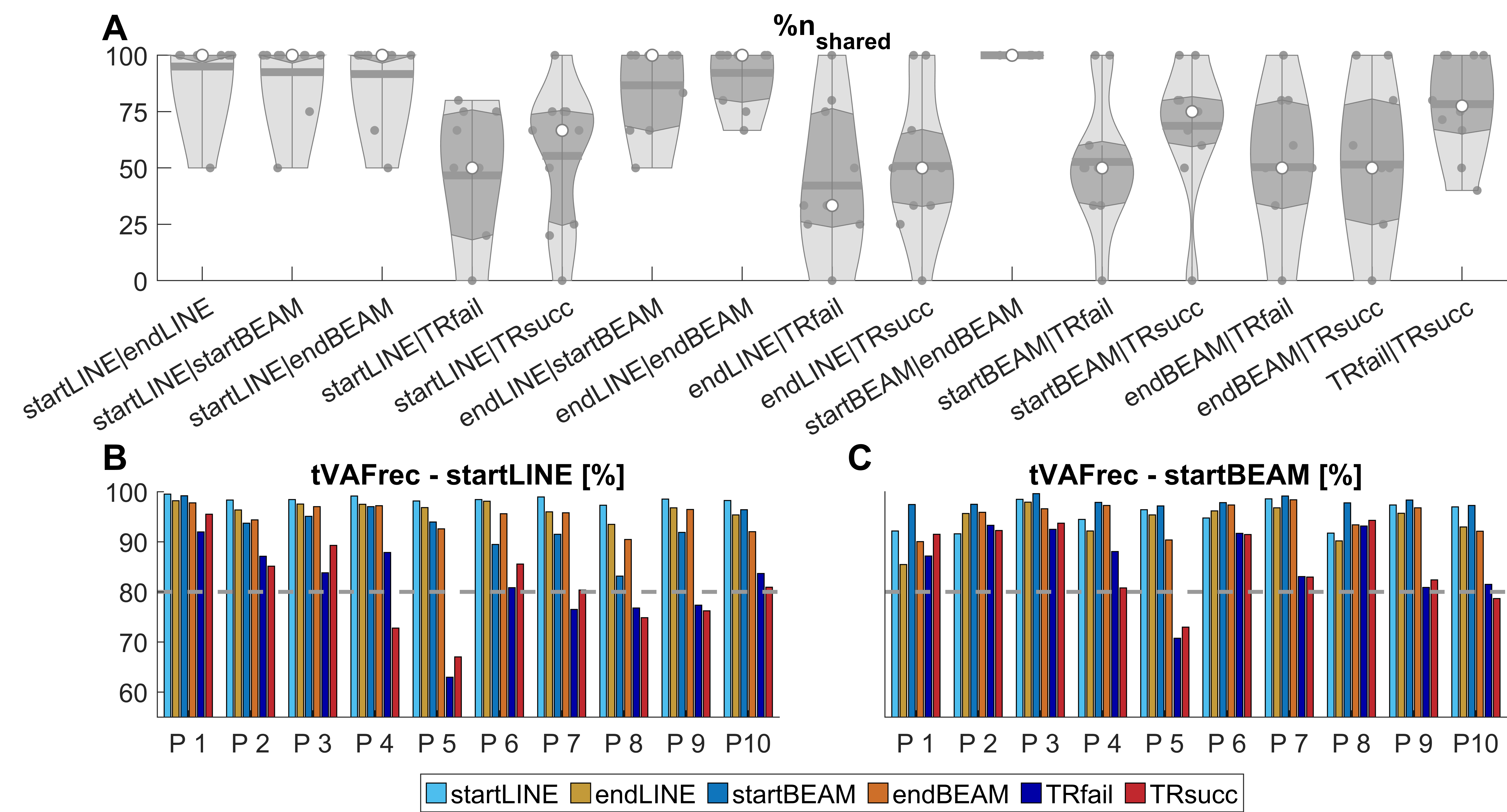


Supplementary Figure S5: A: The percentage of shared synergy weights (n%_shared_) for all possible pairs of condition comparisons. B-C: the tVAF of reconstructed activation coefficients (tVAFrec) of the synergy weights of either startLINE (B) or startBEAM (C) for all participants (P) and conditions. Violin plots: each grey circle represents one participant; thick lines represent mean values; white circles indicate median values; dark areas indicate quartiles.

## Discussion

Here we want to discuss in more detail our approach to extracting synergies across different conditions. Similar tVAFNoS values (main paper) indicate that extracted the extracted synergies equally reflect EMG variability among conditions. However, computing muscle synergies over different conditions has only been done in a few studies so far [20, 24, 38-40]. We used this approach due to the following four considerations: (1) similar movement goals are controlled by similar muscle synergies, (2) the small number of trials within each condition, (3) computing trial-to-trial similarity among similar synergy weights, and (4) the computational problem of extracting accurate synergies when the timing of activation coefficients overlaps.

(1) As mentioned in the introduction, an important feature of motor control is the recruitment of similar synergy weights for similar mechanical goals in different tasks [20, 28, 30, 36, 41, 42]. In the current study, the same movement goal – performing a step – was intended in all conditions. Therefore, we hypothesized that similar synergy weights were used. To verify this assumption, synergies were independently extracted from each condition [20, 39, 40] in an additional analysis. As a first step, we analyzed the percentage of shared synergy weights (r > 0.684 [24-30]). We found that a high percentage of weights was shared between LINE and BEAM conditions. However, fewer synergies were shared when comparing LINE and BEAM conditions to TRfail or TRsucc (Supplementary Figure S5). In a second step, we reconstructed the activation coefficients of all conditions with synergy weights of startLINE or startBEAM. We achieved sufficient reconstruction performance (tVAF > 80% [35-37]) for all LINE and BEAM conditions (Supplementary Figure S5). When reconstructing TIGHTROPE conditions from startLINE, 6 participants achieved a tVAF above 80%, while for startBEAM, it was achieved by 9 participants in TRfail and 8 participants in TRsucc. Turpin et al. [43] revealed that certain synergies were barely activated during low-intensity cycling, which could lead to poor performance of factorization methods in detecting them [2, 44]. Similarly, in our study, we observed differences in the contribution of synergy weights across conditions (see main paper). Cluster 2, which primarily consists of trunk muscles, had a tVAF below 10% for LINE and BEAM conditions but exceeded the threshold for TIGHTROPE. The lower reconstruction accuracy of TIGHTROPE compared to LINE and BEAM conditions may be explained by the fact that these synergies were not captured by extracting synergies separately. Furthermore, cluster 1, which primarily consists of quadriceps and gluteus muscles, exhibited a tVAF below 10% in most LINE synergies but only in some BEAM synergies, which may explain the higher reconstruction accuracies of TIGHTROPE when using startBEAM rather than startLINE. The reconstruction results, combined with cluster analyses, suggest that similar synergy weights are present across tasks, but additional synergies are either added or more activated during balancing tasks. The low percentage of shared synergies in some participants may be due to the difficulty of accurately extracting synergy weights when their activation timing overlaps (see below).

(2) Several studies have emphasized the importance of variability in EMG data through concatenated trials for synergy extraction [2, 45-47]. Oliveira et al. [45] recommended computing muscle synergies over a minimum of 20 concatenated gait-cycles. However, due to the within-session design of the current study, each condition only included four to five stance-phases. Concatenating data from all conditions resulted in a total of 24 to 30 stance phases per participant.

(3) To ensure that the variability of the activation coefficients could be meaningfully quantified, the same synergy weights were used across conditions. This approach aligns with the methodology of Cheung et al. [48], who first clustered the synergy weights across bowling sessions and reconstructed the EMG matrices of each session using the cluster centroids.

(4) Increasing the time overlap (i.e. correlation) of synergy activation coefficients in simulated or real datasets reduces the accuracy of extracted synergy weights. This occurs because, with sufficient coupling, synergy weights merge due to underlying assumptions of factorization algorithms [11, 44, 46]. We believe that the overlap and timing of activation coefficients may reflect an essential feature for movement proficiency and learning. Calculating muscle synergies over all conditions may overcome limitations of extraction algorithms.

This additional analysis showed that similar synergies were used across tasks. Moreover, we hypothesize that calculating synergies over different tasks with similar movement goals (i.e., performing a step) provides salient information in synergy analysis.

# Trial-to-trial similarity of EMG envelopes

## Methods

To determine the overall trial-to-trial similarity of EMG envelopes, we calculated the average value (r, r_max_, lag%) of all pairwise combinations of envelopes from different trials within muscles for each condition. Additionally, correlation values were also averaged for each muscle separately to assess whether similarity differed in each muscle.

Muscle abbreviations: tibialis anterior (tib_abt), peroneus longus (per_long), soleus, gastrocnemius medialis (gast_med), vastus lateralis (vast_lat), rectus femoris (rect_fem), biceps femoris (bic_fem), semitendinosus (sem_tend), gluteus maximus (glut_max), rectus abdominis (rect_abd), extensor obliques (ext_obli), multifidus (multifid) and erector spinae iliocostalis (erec_spin).

## Results

Overall trial-to-trial similarity of EMG envelopes measured by r and r_max_ was significantly affected by TASK (p < 0.001), with LINE showing the highest correlation, followed by BEAM, and TIGHTROPE at last (r LINE vs BEAM: p < 0.01; others: p < 0.001). TIME influenced r (p < 0.01) and r_max_ (p < 0.05) and contrasts revealed lower r and r_max_ (p < 0.05) for startBEAM compared to endBEAM, and an increase in r (p < 0.01) between TRfail and TRsucc. The lag% was significantly affected by TASK (p < 0.01), with higher values in TIGHTROPE compared to LINE (p < 0.01). (Supplementary Figure S6).

**
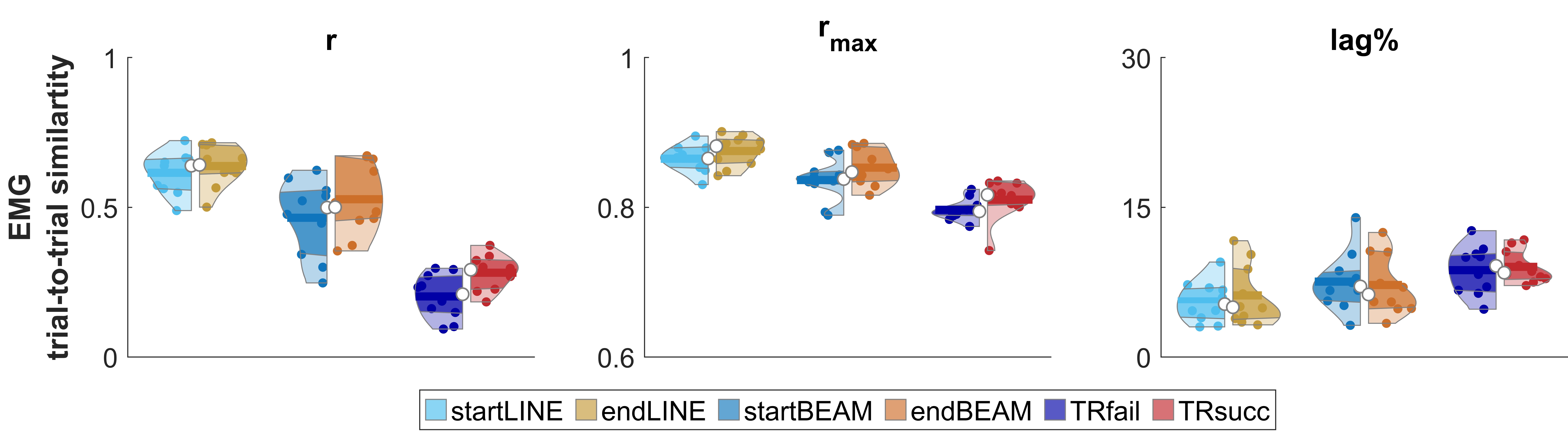
**

Supplementary Figure S6: Overall trial-to-trial similarity of EMG envelopes, measured by Pearson correlation (r), maximum cross-correlation coefficient (r_max_) and lag at r_max_ (lag%). Violin plots: each colored circle represents one participant; thick lines represent mean values; white circles indicate median values; dark areas indicate quartiles.

### Individual muscles

Trial-to-trial similarity in muscle activation patterns measured by Pearson correlation coefficient (r) was significantly affected by TASK in all muscles apart from rec_abd and ext_obli (tib_ant, per_long: p < 0.05; rect_fem: p < 0.01; others: p < 0.001). Trial-to-trial similarity was smaller for TIGHTROPE than BEAM in eight muscles (vast_lat: p < 0.05; soleus, gast_med, bic_fem, sem_tend, glut_max, multifid, erec_spin: p < 0.001), and smaller for TIGHTROPE compared with LINE in all muscles (tib_ant, per_long: p < 0.05; rect_fem: p < 0.01; others: p < 0.001). Trial-to-trial similarity was smaller for BEAM compared to LINE in five muscles (soleus, rect_fem: p < 0.05; multifid: p < 0.01; gast_med, erec_spin: p < 0.001). Additionally, similarity was significantly affected by TIME (per_long, gast_med, glut_max: p < 0.05), and TASK × TIME (gast_med, glut_max: p < 0.05; soleus: p < 0.01) in three muscles. Contrasts revealed higher similarities in endBEAM than startBEAM (gast_med, multifid: p < 0.05, erec_spin: p < 0.01) and TRsucc than TRfail (per_long, gast_med, glut_max: p < 0.05) (Supplementary Table S1).

Trial-to-trial similarity measured by cross-correlation coefficient (r_max_) was significantly affected by TASK in six muscles (soleus, gast_med, glut_max, ext_obli, multifid, erec_spin: p < 0.001), where TIGHTROPE similarity was smaller than BEAM (soleus: p < 0.05; others: p < 0.001) and LINE (p < 0.001). BEAM similarity was smaller than LINE in four muscles (soleus: p < 0.05; gast_med, multifid, erec_spin: p < 0.001). Additionally, similarity was also significantly affected by TIME in four muscles (per_long, soleus, gast_med, multifid, erec_spin: p < 0.05) with higher similarities in END, and TASK × TIME in soleus (p < 0.05). Contrasts revealed higher similarities in endLINE than startLINE (erec_spin: p < 0.05), endBEAM than startBEAM (soleus, gast_med, erec_spin: p < 0.05) and TRsucc than TRfail (multifid: p < 0.05; soleus: p < 0.01) (Supplementary Table S2).

The lag% was significantly affected by TASK for all muscles apart from multifid and erec_spin (tib_ant, per_long, soleus, rec_abd: p < 0.05: gast_med; rect_fem, bic_fem, multifid: p < 0.01; others: p < 0.001). lag% was significantly lower in TIGHTROPE than BEAM for per_long (per_long: p < 0.05). For all other muscles TIGHTROPE had a higher lag% than LINE (tib_ant, gast_med, rec_abd: p < 0.05; soleus, rect_fem, bic_fem, multifid: p < 0.01; others: p < 0.001) and for sem_tend and glut_max also than BEAM (p < 0.001). BEAM had higher lag% than LINE in three muscles (gast_med, bic_fem: p < 0.05; vast_lat: p < 0.01) (Supplementary Table S3).

Supplementary Table S1: Mean (M) and standard deviation (SD) among participants of for trial-to-trial similarity measured by Pearson correlation coefficient r for all conditions and muscles. ANOVA revealed significant effects of TASK in all muscles apart from rec_abd and ext_obli. Significant differences observed by contrasts are indicated by *.

|  | **LINE** | | | | **BEAM** | | | | **TIGHTROPE** | | | |
| --- | --- | --- | --- | --- | --- | --- | --- | --- | --- | --- | --- | --- |
|  | **start** | | **end** | | **start** | | **end** | | **fail** | | **succ** | |
|  | *M* | *SD* | *M* | *SD* | *M* | *SD* | *M* | *SD* | *M* | *SD* | *M* | *SD* |
| tib_ant | 0.49 | 0.22 | 0.60 | 0.17 | 0.43 | 0.15 | 0.45 | 0.20 | 0.36 | 0.19 | 0.36 | 0.21 |
| per_long | 0.49 | 0.22 | 0.61 | 0.20 | 0.45 | 0.16 | 0.48 | 0.22 | 0.31* | 0.16 | 0.44* | 0.19 |
| soleus | 0.84 | 0.07 | 0.80 | 0.10 | 0.64 | 0.18 | 0.67 | 0.17 | 0.23 | 0.19 | 0.31 | 0.18 |
| gast_med | 0.83 | 0.08 | 0.83 | 0.08 | 0.61* | 0.21 | 0.69* | 0.19 | 0.17* | 0.16 | 0.31* | 0.11 |
| vast_lat | 0.78 | 0.20 | 0.73 | 0.28 | 0.56 | 0.23 | 0.60 | 0.20 | 0.31 | 0.18 | 0.41 | 0.16 |
| rect_fem | 0.62 | 0.23 | 0.63 | 0.31 | 0.36 | 0.28 | 0.47 | 0.33 | 0.24 | 0.13 | 0.36 | 0.18 |
| bic_fem | 0.66 | 0.20 | 0.70 | 0.18 | 0.55 | 0.29 | 0.60 | 0.16 | 0.27 | 0.17 | 0.31 | 0.11 |
| sem_tend | 0.63 | 0.27 | 0.76 | 0.17 | 0.62 | 0.24 | 0.65 | 0.18 | 0.23 | 0.14 | 0.26 | 0.16 |
| glut_max | 0.81 | 0.08 | 0.79 | 0.18 | 0.61 | 0.23 | 0.70 | 0.20 | 0.14* | 0.18 | 0.32* | 0.12 |
| rec_abd | 0.15 | 0.23 | 0.02 | 0.12 | 0.13 | 0.20 | 0.08 | 0.09 | 0.11 | 0.07 | 0.11 | 0.14 |
| ext_obli | 0.28 | 0.18 | 0.26 | 0.23 | 0.16 | 0.21 | 0.29 | 0.16 | 0.07 | 0.11 | 0.18 | 0.18 |
| multifid | 0.82 | 0.09 | 0.85 | 0.06 | 0.57* | 0.25 | 0.68* | 0.16 | 0.10 | 0.10 | 0.17 | 0.18 |
| erec_spin | 0.62 | 0.14 | 0.69 | 0.18 | 0.35* | 0.21 | 0.49* | 0.20 | 0.10 | 0.08 | 0.11 | 0.10 |

Supplementary Table S2: Mean (M) and standard deviation (SD) among participants of for trial-to-trial similarity measured by the maximum cross-correlation coefficient r_max_ for all conditions and muscles. ANOVA revealed significant effects of TASK in soleus, gast_med, glut_max, ext_obli, multifid and erec_spin. Significant differences observed by contrasts are indicated by *.

|  | **LINE** | | | | **BEAM** | | | | **TIGHTROPE** | | | |
| --- | --- | --- | --- | --- | --- | --- | --- | --- | --- | --- | --- | --- |
|  | **start** | | **end** | | **start** | | **end** | | **fail** | | **succ** | |
|  | *M* | *SD* | *M* | *SD* | *M* | *SD* | *M* | *SD* | *M* | *SD* | *M* | *SD* |
| tib_ant | 0.81 | 0.06 | 0.83 | 0.06 | 0.85 | 0.04 | 0.86 | 0.04 | 0.87 | 0.04 | 0.87 | 0.07 |
| per_long | 0.88 | 0.05 | 0.90 | 0.04 | 0.87 | 0.05 | 0.87 | 0.06 | 0.88 | 0.02 | 0.90 | 0.03 |
| soleus | 0.96 | 0.02 | 0.95 | 0.02 | 0.91* | 0.04 | 0.93* | 0.03 | 0.87* | 0.04 | 0.90* | 0.03 |
| gast_med | 0.95 | 0.03 | 0.95 | 0.03 | 0.88* | 0.04 | 0.92* | 0.04 | 0.84 | 0.03 | 0.86 | 0.04 |
| vast_lat | 0.91 | 0.05 | 0.89 | 0.08 | 0.85 | 0.08 | 0.87 | 0.06 | 0.86 | 0.05 | 0.86 | 0.06 |
| rect_fem | 0.85 | 0.09 | 0.85 | 0.08 | 0.80 | 0.07 | 0.84 | 0.09 | 0.82 | 0.06 | 0.84 | 0.06 |
| bic_fem | 0.83 | 0.06 | 0.84 | 0.07 | 0.84 | 0.05 | 0.83 | 0.05 | 0.82 | 0.04 | 0.80 | 0.04 |
| sem_tend | 0.86 | 0.07 | 0.89 | 0.05 | 0.85 | 0.08 | 0.86 | 0.03 | 0.83 | 0.05 | 0.80 | 0.07 |
| glut_max | 0.92 | 0.04 | 0.92 | 0.04 | 0.88 | 0.06 | 0.90 | 0.05 | 0.77 | 0.06 | 0.79 | 0.08 |
| rec_abd | 0.72 | 0.07 | 0.72 | 0.06 | 0.74 | 0.07 | 0.72 | 0.05 | 0.69 | 0.04 | 0.71 | 0.08 |
| ext_obli | 0.82 | 0.04 | 0.82 | 0.04 | 0.81 | 0.05 | 0.82 | 0.03 | 0.72 | 0.05 | 0.76 | 0.05 |
| multifid | 0.90 | 0.05 | 0.92 | 0.03 | 0.83 | 0.07 | 0.86 | 0.05 | 0.72* | 0.06 | 0.76* | 0.07 |
| erec_spin | 0.83* | 0.05 | 0.89* | 0.07 | 0.76* | 0.08 | 0.80* | 0.07 | 0.67 | 0.03 | 0.68 | 0.07 |

Supplementary Table S3: Mean (M) and standard deviation (SD) among participants of for trial-to-trial similarity measured by the lag time lag% at the maximum cross-correlation coefficient for all conditions and muscles. ANOVA revealed significant effects of TASK in all muscles apart from multifid and erec_spin.

|  | **LINE** | | | | **BEAM** | | | | **TIGHTROPE** | | | |
| --- | --- | --- | --- | --- | --- | --- | --- | --- | --- | --- | --- | --- |
|  | **start** | | **end** | | **start** | | **end** | | **fail** | | **succ** | |
|  | *M* | *SD* | *M* | *SD* | *M* | *SD* | *M* | *SD* | *M* | *SD* | *M* | *SD* |
| tib_ant | 6.06 | 7.58 | 5.55 | 5.02 | 5.60 | 4.01 | 6.64 | 4.88 | 9.26 | 5.93 | 10.24 | 7.67 |
| per_long | 6.84 | 3.79 | 5.54 | 4.41 | 7.62 | 3.88 | 7.06 | 3.44 | 5.13 | 3.06 | 3.82 | 2.20 |
| soleus | 1.98 | 0.63 | 2.95 | 1.46 | 4.58 | 1.11 | 3.66 | 2.13 | 6.95 | 4.92 | 6.09 | 3.09 |
| gast_med | 2.94 | 1.18 | 2.72 | 0.81 | 5.63 | 2.33 | 4.39 | 1.76 | 6.68 | 4.49 | 6.56 | 3.79 |
| vast_lat | 2.89 | 3.32 | 3.39 | 7.39 | 7.09 | 7.59 | 5.04 | 3.76 | 6.93 | 2.86 | 7.99 | 4.22 |
| rect_fem | 3.30 | 3.89 | 6.44 | 11.24 | 7.06 | 7.24 | 9.08 | 11.58 | 10.93 | 3.64 | 8.76 | 4.60 |
| bic_fem | 7.43 | 9.48 | 5.74 | 10.69 | 5.51 | 6.17 | 5.98 | 5.98 | 6.60 | 3.78 | 12.33 | 5.80 |
| sem_tend | 3.92 | 5.89 | 3.10 | 5.58 | 5.23 | 8.82 | 1.63 | 1.83 | 8.37 | 5.12 | 9.17 | 5.52 |
| glut_max | 2.16 | 1.10 | 3.19 | 3.85 | 5.20 | 4.17 | 5.33 | 6.02 | 13.76 | 4.57 | 11.47 | 7.26 |
| rec_abd | 17.52 | 8.52 | 20.07 | 5.64 | 16.26 | 8.22 | 17.80 | 8.88 | 11.81 | 3.78 | 12.80 | 6.11 |
| ext_obli | 8.43 | 6.32 | 8.07 | 5.82 | 7.45 | 3.80 | 8.62 | 5.34 | 8.63 | 5.16 | 9.14 | 6.11 |
| multifid | 0.90 | 0.61 | 3.78 | 6.61 | 4.95 | 5.35 | 4.32 | 7.74 | 6.98 | 4.70 | 6.22 | 6.70 |
| erec_spin | 7.94 | 8.33 | 10.15 | 14.38 | 15.95 | 12.86 | 14.14 | 17.25 | 11.02 | 7.92 | 12.78 | 8.24 |

# Results of individual joints

Joint abbrevations: ankle plantar-/dorsiflexion (ankle_flex), knee flexion/extension (knee_flex), hip flexion/extension (hip_flex), hip ab-/adduction (hip_ad), hip internal/external rotation (hip_rot), lumbar flexion/extension (lumb_flex), lumbar medial/lateral bending (lumb_bend), and lumbar internal/external rotation (lumb_rot).

Trial-to-trial similarity measured by Pearson correlation coefficient (r) was significantly affected in all joints by TASK (ankle_flex: p < 0.01; others: p < 0.001). TIGHTROPE similarity was always lower than BEAM (ankle_flex; p < 0.05; others: p < 0.001) and LINE (ankle_flex: p < 0.01; others: p < 0.001). BEAM similarity was lower than LINE in three joints (knee_flex, hip_ad: p < 0.05; lumb_bend: p < 0.001). It was also significantly affected by TIME in some joints (hip_ad, hip_rot, lumb_rot: p < 0.05; ankle_flex, hip_flex: p < 0.01), with lower similarity in START than END. Additionally, a significant effect of TIME × TASK was found in two joints (hip_rot: p < 0.01; hip_flex: p < 0.001). Contrasts showed that r was lower in startLINE than endLINE (hip_ad: p < 0.05) and TRfail than TRsucc (ankle_flex: p < 0.05) (Supplementary Table S4).

Trial-to-trial similarity measured by cross-correlation coefficient (r_max_) was significantly affected by TASK in all joints (lumb_flex: p < 0.05; hip_flex, hip_rot: p < 0.05; others: p < 0.001). TIGHTROPE similarity was lower than BEAM in six joints (ankle_flex, lumb_flex, lumb_bend: p < 0.05; knee_flex, hip_ad, lumb_rot: p < 0.001) and LINE in all joints (hip_flex, hip_rot: p < 0.05; lumb_flex: p < 0.01; othert: p < 0.001). BEAM similarity was lower than LINE in lumb_bend (p < 0.01). It was also significantly affected by TIME in two joints (lumb_rot: p < 0.01; lumb_flex: p < 0.001), with lower similarity in START than END. Additionally, a significant effect of TIME × TASK was found in lumb_rot (p < 0.05). Contrasts showed that r_max_ was lower in startBEAM than endBEAM (lumb_flex, lumb_bend: p < 0.05) and TRfail than TRsucc (lumb_flex: p < 0.01) (Supplementary Table S5).

The lag% was significantly affected by TASK in five joints (hip_rot, lumb_flex: p < 0.01; hip_ad, lumb_bend, lumb_rot: p < 0.001). TIGHTROPE had a higher lag% than BEAM in four joints (hip_ad, hip_rot, lumb_rot: p < 0.01; lumb_bend: p < 0.001) and LINE in all five joints (hip_rot, lumb_rot: p < 0.01; other: p < 0.001). BEAM had a higher lag% than LINE in two jonts (hip_ad: p < 0.05; lumb_bend: p < 0.001). There was a significant effect of TIME in five joints (hip_ad, lumb_flex, lumb_rot: p < 0.01; knee_flex, hip_rot: p < 0.001) with higher lag% in START. Additionally, there was a significant effect of TASK × TIME in three joints (knee_flex, hip_ad: p < 0.01; hip_rot: p < 0.001). Contrasts revealed higher lag% in startLINE than endLINE in hip_ad (p < 0.05). For lumb_flex, startBEAM and TRfail had significantly higher lag% compared to endBEAM and TRsucc (p < 0.01), respectively (Supplementary Table S6).

Supplementary Table S4: Mean (M) and standard deviation (SD) among participants of for trial-to-trial similarity measured by Pearson correlation coefficient r for all conditions and joint angles. ANOVA revealed significant effects of TASK in all joints Significant differences observed by contrasts are indicated by *.

|  | **LINE** | | | | **BEAM** | | | | **TIGHTROPE** | | | |
| --- | --- | --- | --- | --- | --- | --- | --- | --- | --- | --- | --- | --- |
|  | **start** | | **end** | | **start** | | **end** | | **fail** | | **succ** | |
|  | *M* | *SD* | *M* | *SD* | *M* | *SD* | *M* | *SD* | *M* | *SD* | *M* | *SD* |
| ankle_flex | 0.95 | 0.04 | 0.97 | 0.02 | 0.92 | 0.07 | 0.94 | 0.06 | 0.77* | 0.21 | 0.88* | 0.06 |
| knee_flex | 0.97 | 0.02 | 0.97 | 0.02 | 0.93 | 0.07 | 0.91 | 0.07 | 0.53 | 0.31 | 0.54 | 0.27 |
| hip_flex | 0.96 | 0.10 | 0.99 | 0.01 | 0.95 | 0.14 | 0.97 | 0.04 | 0.66 | 0.32 | 0.88 | 0.12 |
| hip_ab | 0.89* | 0.15 | 0.96* | 0.02 | 0.69 | 0.35 | 0.76 | 0.28 | 0.19 | 0.20 | 0.31 | 0.20 |
| hip_rot | 0.94 | 0.13 | 0.97 | 0.01 | 0.90 | 0.20 | 0.96 | 0.05 | 0.55 | 0.36 | 0.76 | 0.17 |
| lumb_flex | 0.71 | 0.18 | 0.69 | 0.24 | 0.46 | 0.28 | 0.56 | 0.31 | 0.01 | 0.17 | 0.22 | 0.28 |
| lumb_bend | 0.88 | 0.16 | 0.90 | 0.15 | 0.40 | 0.33 | 0.56 | 0.32 | 0.08 | 0.18 | -0.04 | 0.21 |
| lumb_rot | 0.93 | 0.14 | 0.98 | 0.02 | 0.82 | 0.26 | 0.88 | 0.26 | 0.31 | 0.29 | 0.38 | 0.38 |

Supplementary Table S5: Mean (M) and standard deviation (SD) among participants of for trial-to-trial similarity measured by the maximum cross-correlation coefficient r_max_ for all conditions and joint angles. ANOVA revealed significant effects of TASK in all joints Significant differences observed by contrasts are indicated by *.

|  | **LINE** | | | | **BEAM** | | | | **TIGHTROPE** | | | |
| --- | --- | --- | --- | --- | --- | --- | --- | --- | --- | --- | --- | --- |
|  | **start** | | **end** | | **start** | | **end** | | **fail** | | **succ** | |
|  | *M* | *SD* | *M* | *SD* | *M* | *SD* | *M* | *SD* | *M* | *SD* | *M* | *SD* |
| ankle_flex | 0.94 | 0.05 | 0.97 | 0.01 | 0.89 | 0.11 | 0.93 | 0.05 | 0.73 | 0.18 | 0.81 | 0.15 |
| knee_flex | 0.97 | 0.06 | 0.98 | 0.01 | 0.96 | 0.08 | 0.97 | 0.04 | 0.95 | 0.04 | 0.96 | 0.03 |
| hip_flex | 0.96 | 0.08 | 0.99 | 0.01 | 0.94 | 0.13 | 0.97 | 0.03 | 0.95 | 0.03 | 0.96 | 0.03 |
| hip_ab | 0.97 | 0.03 | 0.99 | 0.01 | 0.92 | 0.07 | 0.90 | 0.13 | 0.56 | 0.19 | 0.66 | 0.14 |
| hip_rot | 0.90 | 0.13 | 0.96 | 0.04 | 0.87 | 0.14 | 0.93 | 0.04 | 0.78 | 0.19 | 0.86 | 0.17 |
| lumb_flex | 0.83 | 0.20 | 0.90 | 0.17 | 0.79* | 0.19 | 0.91* | 0.14 | 0.61* | 0.17 | 0.80* | 0.16 |
| lumb_bend | 0.85 | 0.08 | 0.88 | 0.15 | 0.65* | 0.18 | 0.68* | 0.19 | 0.49 | 0.09 | 0.49 | 0.13 |
| lumb_rot | 0.92 | 0.07 | 0.95 | 0.03 | 0.82 | 0.16 | 0.88 | 0.15 | 0.50 | 0.20 | 0.65 | 0.21 |

Supplementary Table S6: Mean (M) and standard deviation (SD) among participants of for trial-to-trial similarity measured by the lag time lag% at the maximum cross-correlation coefficient for all conditions and joint angles. ANOVA revealed significant effects of TASK in hip_rot, hip_ad, lumb_bend, lumb_flex. Significant differences observed by contrasts are indicated by *.

|  | **LINE** | | | | **BEAM** | | | | **TIGHTROPE** | | | |
| --- | --- | --- | --- | --- | --- | --- | --- | --- | --- | --- | --- | --- |
|  | **start** | | **end** | | **start** | | **end** | | **fail** | | **succ** | |
|  | *M* | *SD* | *M* | *SD* | *M* | *SD* | *M* | *SD* | *M* | *SD* | *M* | *SD* |
| ankle_flex | 0.22 | 0.44 | 0.00 | 0.00 | 4.35 | 12.20 | 1.04 | 2.85 | 8.75 | 18.91 | 3.18 | 8.21 |
| knee_flex | 0.00 | 0.00 | 0.00 | 0.00 | 0.00 | 0.00 | 0.00 | 0.00 | 0.03 | 0.09 | 0.00 | 0.00 |
| hip_flex | 2.76 | 8.73 | 0.00 | 0.00 | 2.00 | 6.32 | 0.00 | 0.00 | 0.89 | 1.86 | 0.23 | 0.74 |
| hip_ab | 2.42* | 4.79 | 0.55* | 0.56 | 7.51 | 9.51 | 7.75 | 11.83 | 32.02 | 15.47 | 21.63 | 10.85 |
| hip_rot | 3.82 | 9.94 | 0.22 | 0.40 | 4.68 | 12.77 | 0.86 | 1.17 | 13.14 | 13.23 | 3.30 | 8.99 |
| lumb_flex | 10.92 | 15.24 | 4.03 | 10.93 | 17.89* | 17.68 | 4.44* | 11.05 | 24.66* | 16.25 | 11.48* | 11.94 |
| lumb_bend | 2.62 | 5.32 | 5.71 | 14.86 | 19.90 | 14.77 | 19.23 | 17.12 | 40.14 | 8.57 | 44.70 | 12.60 |
| lumb_rot | 2.55 | 6.77 | 0.49 | 1.05 | 8.85 | 13.36 | 4.74 | 11.61 | 31.68 | 18.03 | 18.96 | 14.74 |

# References

[1] M. Ghislieri, M. Lanotte, M. Knaflitz, L. Rizzi, and V. Agostini, "Muscle synergies in Parkinson’s disease before and after the deep brain stimulation of the bilateral subthalamic nucleus," *Sci Rep,* vol. 13, p. 6997, 2023, doi: 10.1038/s41598-023-34151-6.

[2] N. A. Turpin, S. Uriac, and G. Dalleau, "How to improve the muscle synergy analysis methodology?," *Eur J Appl Physiol,* vol. 121, p. 1025, 2021, doi: 10.1007/s00421-021-04604-9.

[3] V. L. S. Profeta and M. T. Turvey, "Bernstein’s levels of movement construction: A contemporary perspective," *Hum Mov Sci,* vol. 57, p. 133, 2018, doi: 10.1016/j.humov.2017.11.013.

[4] H. S. Seung and D. D. Lee, "Learning the parts of objects by non-negative matrix factorization," *Nature,* vol. 401, p. 791, 1999, doi: 10.1038/44565.

[5] D. Lee and H. Seung, "Algorithms for Non-negative Matrix Factorization," *Adv. Neural Inform. Process. Syst.,* vol. 13, 02/10 2001.

[6] P. Paatero and U. Tapper, "Positive matrix factorization: A non-negative factor model with optimal utilization of error estimates of data value," *Environmetrics,* vol. 5, pp. 111-126, 1994.

[7] Y. Kim, T. C. Bulea, and D. L. Damiano, "Novel Methods to Enhance Precision and Reliability in Muscle Synergy Identification during Walking," *Front Hum Neurosci,* vol. 10, p. 455, 2016, doi: 10.3389/fnhum.2016.00455.

[8] C. Boutsidis and E. Gallopoulos, "SVD based initialization: A head start for nonnegative matrix factorization," *Pattern recognition,* vol. 41, p. 1362, 2008, doi: 10.1016/j.patcog.2007.09.010.

[9] S. M. Atif, S. Qazi, and N. Gillis, "Improved SVD-based initialization for nonnegative matrix factorization using low-rank correction," *Pattern recognition letters,* vol. 122, p. 59, 2019, doi: 10.1016/j.patrec.2019.02.018.

[10] Z. Zheng, J. Yang, and Y. Zhu, "Initialization enhancer for non-negative matrix factorization," *Engineering applications of artificial intelligence,* vol. 20, p. 110, 2007, doi: 10.1016/j.engappai.2006.03.001.

[11] M. H. Soomro, S. Conforto, G. Giunta, S. Ranaldi, and C. De Marchis, "Comparison of Initialization Techniques for the Accurate Extraction of Muscle Synergies from Myoelectric Signals via Nonnegative Matrix Factorization," *Appl Bionics Biomech,* vol. 2018, p. 10, 2018, doi: 10.1155/2018/3629347.

[12] J. F. Yang and M. Gorassini, "Spinal and brain control of human walking: implications for retraining of walking," *Neuroscientist,* vol. 12, p. 389, 2006, doi: 10.1177/1073858406292151.

[13] F. Sylos-Labini *et al.*, "Complexity of modular neuromuscular control increases and variability decreases during human locomotor development," *Communications Biology,* vol. 5, no. 1, p. 1256, 2022/11/16 2022, doi: 10.1038/s42003-022-04225-8.

[14] Y. P. Ivanenko *et al.*, "Changes in the spinal segmental motor output for stepping during development from infant to adult," *J Neurosci,* vol. 33, p. 36a, 2013, doi: 10.1523/JNEUROSCI.2722-12.2013.

[15] F. Hug, "Can muscle coordination be precisely studied by surface electromyography?," *J Electromyogr Kinesiol,* vol. 21, p. 12, 2011, doi: 10.1016/j.jelekin.2010.08.009.

[16] F. Hug, N. A. Turpin, S. Dorel, and A. Guével, "Smoothing of electromyographic signals can influence the number of extracted muscle synergie," *Clin Neurophysiol,* vol. 123, p. 1896, 2012, doi: 10.1016/j.clinph.2012.01.015.

[17] M. van der Krogt, L. Oudenhoven, A. Buizer, A. Dallmeijer, N. Dominici, and J. Harlaar, "The effect of EMG processing choices on muscle synergies before and after BoNT-A treatment in cerebral palsy," *Gait & posture,* vol. 49, p. 31, 2016, doi: 10.1016/j.gaitpost.2016.07.095.

[18] B. R. Shuman, M. H. Schwartz, and K. M. Steele, "Electromyography Data Processing Impacts Muscle Synergies during Gait for Unimpaired Children and Children with Cerebral Palsy," (in English), *Frontiers in Computational Neuroscience,* Original Research vol. 11, no. 50, 2017-June-06 2017, doi: 10.3389/fncom.2017.00050.

[19] P. Kieliba, P. Tropea, E. Pirondini, M. Coscia, S. Micera, and F. Artoni, "How Are Muscle Synergies Affected by Electromyography Pre-Processing?," *IEEE Trans Neural Syst Rehabil Eng,* vol. 26, p. 893, 2018, doi: 10.1109/TNSRE.2018.2810859.

[20] F. Hug, N. A. Turpin, A. Couturier, and S. Dorel, "Consistency of muscle synergies during pedaling across different mechanical constraints," (in eng), *J Neurophysiol,* vol. 106, no. 1, pp. 91-103, Jul 2011, doi: 10.1152/jn.01096.2010.

[21] F. Hug *et al.*, "Individuals have unique muscle activation signatures as revealed during gait and pedaling," *J Appl Physiol (1985),* vol. 127, p. 1174, 2019, doi: 10.1152/japplphysiol.01101.2018.

[22] A. J. Meyer, I. Eskinazi, J. N. Jackson, A. V. Rao, C. Patten, and B. J. Fregly, "Muscle Synergies Facilitate Computational Prediction of Subject-Specific Walking Motions," *Front Bioeng Biotechnol,* vol. 4, p. 77, 2016, doi: 10.3389/fbioe.2016.00077.

[23] C. L. Banks, M. M. Pai, T. E. McGuirk, B. J. Fregly, and C. Patten, "Methodological choices in muscle synergy analysis impact differentiation of physiological characteristics following stroke," *Front Comput Neurosci,* vol. 11, p. 78, 2017, doi: 10.3389/fncom.2017.00078.

[24] S. A. Safavynia and L. H. Ting, "Task-level feedback can explain temporal recruitment of spatially fixed muscle synergies throughout postural perturbations," *J Neurophysiol,* vol. 107, p. 177, 2012, doi: 10.1152/jn.00653.2011.

[25] K. Zhao, Z. Zhang, H. Wen, and A. Scano, "Intra-Subject and Inter-Subject Movement Variability Quantified with Muscle Synergies in Upper-Limb Reaching Movements," *Biomimetics (Basel, Switzerland),* vol. 6, 2021, doi: 10.3390/biomimetics6040063.

[26] J. L. Allen, J. L. McKay, A. Sawers, M. E. Hackney, and L. H. Ting, "Increased neuromuscular consistency in gait and balance after partnered, dance-based rehabilitation in parkinson’s disease," *J Neurophysiol,* vol. 118, p. 373, 2017, doi: 10.1152/jn.00813.2016.

[27] J. L. Allen, T. M. Kesar, and L. H. Ting, "Motor module generalization across balance and walking is impaired after stroke," *J Neurophysiol,* vol. 122, p. 289, 2019, doi: 10.1152/jn.00561.2018.

[28] J. L. Allen, H. D. Carey, L. H. Ting, and A. Sawers, "Generalization of motor module recruitment across standing reactive balance and walking is associated with beam walking performance in young adults," *Gait Posture,* vol. 82, p. 247, 2020, doi: 10.1016/j.gaitpost.2020.09.016.

[29] J. Frère and F. Hug, "Between-subject variability of muscle synergies during a complex motor skill," (in English), *Frontiers in Computational Neuroscience,* Original Research vol. 6, no. 99, 2012-December-28 2012, doi: 10.3389/fncom.2012.00099.

[30] S. A. Chvatal, G. Torres-Oviedo, S. A. Safavynia, and L. H. Ting, "Common muscle synergies for control of center of mass and force in nonstepping and stepping postural behaviors," *J Neurophysiol,* vol. 106, p. 1015, 2011, doi: 10.1152/jn.00549.2010.

[31] M. Kristiansen, A. Samani, P. Madeleine, and E. A. Hansen, "Muscle synergies during bench press are reliable across days," *J Electromyogr Kinesiol,* vol. 30, p. 88, 2016, doi: 10.1016/j.jelekin.2016.06.004.

[32] S. Muceli, A. T. Boye, A. D'Avella, and D. Farina, "Identifying representative synergy matrices for describing muscular activation patterns during multidirectional reaching in the horizontal plane," *J Neurophysiol,* vol. 103, p. 1542, 2010, doi: 10.1152/jn.00559.2009.

[33] L. Gizzi, J. F. Nielsen, F. Felici, Y. P. Ivanenko, and D. Farina, "Impulses of activation but not motor modules are preserved in the locomotion of subacute stroke patients," *J Neurophysiol,* vol. 106, p. 210, 2011, doi: 10.1152/jn.00727.2010.

[34] W. van den Hoorn, J. H. van Dieen, P. W. Hodges, and F. Hug, "Effect of acute noxious stimulation to the leg or back on muscle synergies during walking," *J Neurophysiol,* vol. 113, p. 54, 2015, doi: 10.1152/jn.00557.2014.

[35] G. Boccia, C. Zoppirolli, L. Bortolan, F. Schena, and B. Pellegrini, "Shared and task‐specific muscle synergies of Nordic walking and conventional walking," *Scand J Med Sci Sports,* vol. 28, p. 918, 2018, doi: 10.1111/sms.12992.

[36] A. S. Oliveira, L. Gizzi, S. Ketabi, D. Farina, and U. G. Kersting, "Modular Control of Treadmill vs Overground Running," *PLoS One,* vol. 11, p. e0153307, 2016, doi: 10.1371/journal.pone.0153307.

[37] L. J. Heales, F. Hug, D. A. MacDonald, B. Vicenzino, and P. W. Hodges, "Is synergistic organisation of muscle coordination altered in people with lateral epicondylalgia? A case–control study," *Clin Biomech (Bristol, Avon),* vol. 35, p. 131, 2016, doi: 10.1016/j.clinbiomech.2016.04.017.

[38] J. Roh, W. Z. Rymer, and R. F. Beer, "Robustness of muscle synergies underlying three-dimensional force generation at the hand in healthy humans," *J Neurophysiol,* vol. 107, p. 2142, 2012, doi: 10.1152/jn.00173.2011.

[39] S. Hagio, M. Fukuda, and M. Kouzaki, "Identification of muscle synergies associated with gait transition in humans," *Front Hum Neurosci,* vol. 9, p. 48, 2015, doi: 10.3389/fnhum.2015.00048.

[40] S. A. Chvatal and L. H. Ting, "Common muscle synergies for balance and walking," *Front Comput Neurosci,* vol. 7, p. 48, 2013, doi: 10.3389/fncom.2013.00048.

[41] M. M. Nazifi, H. U. Yoon, K. Beschorner, and P. Hur, "Shared and task-specific muscle synergies during normal walking and slipping," *Front Hum Neurosci,* vol. 11, p. 40, 2017, doi: 10.3389/fnhum.2017.00040.

[42] F. O. Barroso *et al.*, "Shared muscle synergies in human walking and cycling," *J Neurophysiol,* vol. 112, p. 1998, 2014, doi: 10.1152/jn.00220.2014.

[43] N. A. Turpin, A. Costes, P. Moretto, and B. Watier, "Can muscle coordination explain the advantage of using the standing position during intense cycling?," *J Sci Med Sport,* vol. 20, p. 616, 2017, doi: 10.1016/j.jsams.2016.10.019.

[44] M. C. Tresch, V. C. K. Cheung, and A. d'Avella, "Matrix Factorization Algorithms for the Identification of Muscle Synergies: Evaluation on Simulated and Experimental Data Sets," *J Neurophysiol,* vol. 95, p. 2212, 2006, doi: 10.1152/jn.00222.2005.

[45] A. S. Oliveira, L. Gizzi, D. Farina, and U. G. Kersting, "Motor modules of human locomotion: influence of EMG averaging, concatenation, and number of step cycles," (in English), *Frontiers in Human Neuroscience,* Original Research vol. 8, 2014-May-23 2014, doi: 10.3389/fnhum.2014.00335.

[46] K. M. Steele, M. C. Tresch, and E. J. Perreault, "Consequences of biomechanically constrained tasks in the design and interpretation of synergy analyses," *J Neurophysiol,* vol. 113, p. 2113, 2015, doi: 10.1152/jn.00769.2013.

[47] R. Ranganathan and C. Krishnan, "Extracting synergies in gait: using EMG variability to evaluate control strategies," *J Neurophysiol,* vol. 108, p. 1544, 2012, doi: 10.1152/jn.01112.2011.

[48] V. C. K. Cheung, X.-C. Zheng, R. T. H. Cheung, and R. H. M. Chan, "Modulating the Structure of Motor Variability for Skill Learning Through Specific Muscle Synergies in Elderlies and Young Adults," *IEEE Open J Eng Med Biol,* vol. 1, p. 40, 2020, doi: 10.1109/OJEMB.2019.2963666.

# 
